# Burst-by-Burst Measurement of Rotational Diffusion at Nanosecond Resolution Reveals Hot-Brownian Motion and Single-Chain Binding

**DOI:** 10.1021/acsnano.3c03392

**Published:** 2023-06-23

**Authors:** Nasrin Asgari, Martin Dieter Baaske, Michel Orrit

**Affiliations:** † Huygens-Kamerlingh Onnes Laboratory, Leiden University, Postbus 9504, 2300 RA Leiden, The Netherlands; ‡ Max Planck Institute of Biophysics, Max-von-Laue-Str. 3, 60438 Frankfurt am Main, Germany

**Keywords:** plasmonics, dark-field scattering, biosensing, rotational diffusion, gold nanorods, hot Brownian motion, polymer adsorption

## Abstract

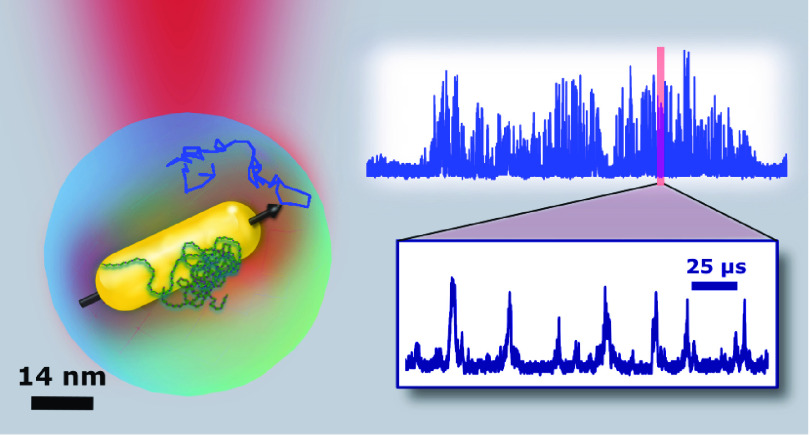

We record dark-field scattering bursts of individual gold nanorods, 52 × 15 nm^2^ in average size, freely diffusing in water suspension. We deduce their Brownian rotational diffusion constant from autocorrelation functions on a single-event basis. Due to spectral selection by the plasmonic resonance with the excitation laser, the distribution of rotational diffusion constants is much narrower than expected from the size distribution measured by TEM. As rotational diffusion depends on particle hydrodynamic volume, viscosity, and temperature, it can sense those parameters at the single-particle level. We demonstrate measurements of hot Brownian rotational diffusion of nanorods in temperature and viscosity gradients caused by plasmonic heating. Further, we monitor hydrodynamic volumes of gold nanorods upon addition of very low concentrations of the water-soluble polymer PVA, which binds to the particles, leading to measurable changes in their diffusion constant corresponding to binding of one to a few polymer coils. We propose this analysis technique for very low concentrations of biomolecules in solution.

## Introduction

After the real-time observation of the rotational diffusion of dye molecules in 1976 by Fleming et al.,^1^ rotational Brownian dynamics had a vast impact in fields ranging from physical chemistry to biology. A traditional and conventional way to monitor the rotational diffusion of (bio)molecules is fluorescence depolarization, which requires fluorescent labeling and separation of rotational diffusion contributions from those of other fast processes, notably photoblinking and photobleaching.^2,3^ Because of these complications, a direct observation of rotational diffusion through scattering is often preferred. In 1990, dynamic light scattering has been used by Pecora^4^ to measure the rotational diffusion of rod-like oligonucleotides in an ensemble measurement. Whenever the tumbling particles are heterogeneous in size or shape or whenever the local environment is inhomogeneous (as in a cell, for example), it is a big advantage to monitor the rotational dynamics of single objects with high time resolution. This requires strong anisotropic scatterers such as plasmonic nanoparticles. Gold nanorods (GNRs) are such strong anisotropic plasmonic scatterers that are chemically inert and very photostable at the low intensities required for scattering measurements. In 2004, Sönnichsen et al. measured the orientation changes of single GNRs in two dimensions.^5^ Later work has studied the tumbling of GNRs in two and three dimensions.^6−10^ To our knowledge, the highest temporal resolution achieved to-date is 2.3 μs for 71 × 25 nm^2^ GNRs on a lipid bilayer^11^ and 3.3 μs for 80 × 40 nm^2^ GNRs on a glass substrate.^12^

For a small enough particle diffusing through the confocal volume of a microscope, the contributions of rotational diffusion and of translational diffusion to the optical signal’s autocorrelation function can be separated due to their very different time scales.^13−15^ For particles a few tens of nanometers in size in water, the translational diffusion in the confocal volume is in the millisecond domain, whereas the rotational diffusion is in the microsecond domain. Neglecting rotational-translational coupling according to Kask et al.^3^ and Widengren et al.,^16^ these contributions to the autocorrelation function of intensity fluctuations can thus be separated as: *G*(τ) = *G*_*T*_(τ)*G*_*R*_(τ), where *G*_*T*_(τ) and *G*_*R*_(τ) are the translational and rotational correlation functions, respectively. The Brownian rotational diffusion coefficient Θ of a rigid cylinder with flat ends can be expressed according to Tirado et al.^17^ as:
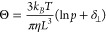
1where *p* = *L*/*D* is the cylinder’s aspect ratio, *L* its length, *D* its diameter, and δ_⊥_ = −0.662 + 0.917/*p* – 0.050/*p*^2^ is an end effect correction. The rotational diffusion coefficient reports on the local temperature and temperature-dependent viscosity of the particle’s environment. As an interesting application, rotational diffusion can be used to explore the effective viscosity of the environment, a method known as nanorheology.^18−21^ Micro- or nanorheology measures the viscoelastic properties of a medium in small volumes from picoliters with microparticles to attoliters with nanoparticles. It has been applied to the study of complex fluids including biological polymers, live cells and media with inhomogeneous mechanical properties.^22,23^

Single-particle microrheology is most often based on tracking translational diffusion of a spherical diffuser.^24^ In a similar way, rotational diffusion can reveal changes of the local conditions, either those due to local heterogeneities, or due to temperature changes in the vicinity of a heat source,^25^ for example. The case where the heat source is the diffuser itself is referred to as (rotational) hot Brownian motion.^26,27^ Moreover, the rotational diffusion coefficient Θ scales with the hydrodynamic volume of the diffuser, (Θ ∝ 1/*L*^3^),^17,28^ and is thus more sensitive to the diffuser’s size than its translational coefficient, which scales as size (*D*_*t*_ ∝ 1/*L*).^29^ Therefore, it is promising to monitor the rotational diffusion of a diffuser to study its conformational changes or the binding of ligand molecules to the diffuser.^30^

In the present work, we measure the polarization-sensitive scattering of single GNRs, 52 × 15 nm^2^ in average size, with high (nanosecond) time resolution. Each scattering event (burst) is determined by an individual nanoparticle crossing the microscope’s confocal volume. During each of those events, the tumbling particle reorients many times, giving rise to hundreds of sub-bursts, which provide a statistically significant sampling of the rotational diffusion rate of each particle on an event-by-event (burst-by-burst) basis. We studied the effect of the polarization configuration on the rotational correlation. By varying the laser intensity, we investigate the change in tumbling rate due to hot Brownian rotation of the nanorods. As another application, we study how tumbling of these particles changes in the presence of very low (tens of ppb to 100 ppm) concentrations of a polymer (PVA) in the solution due to binding of polymer molecules. We find that this process is described by a Langmuir isotherm with evidence of heterogeneity of the binding sites.

## Results and Discussion

### Rotational Diffusion and Scattering

**Figure 1:**
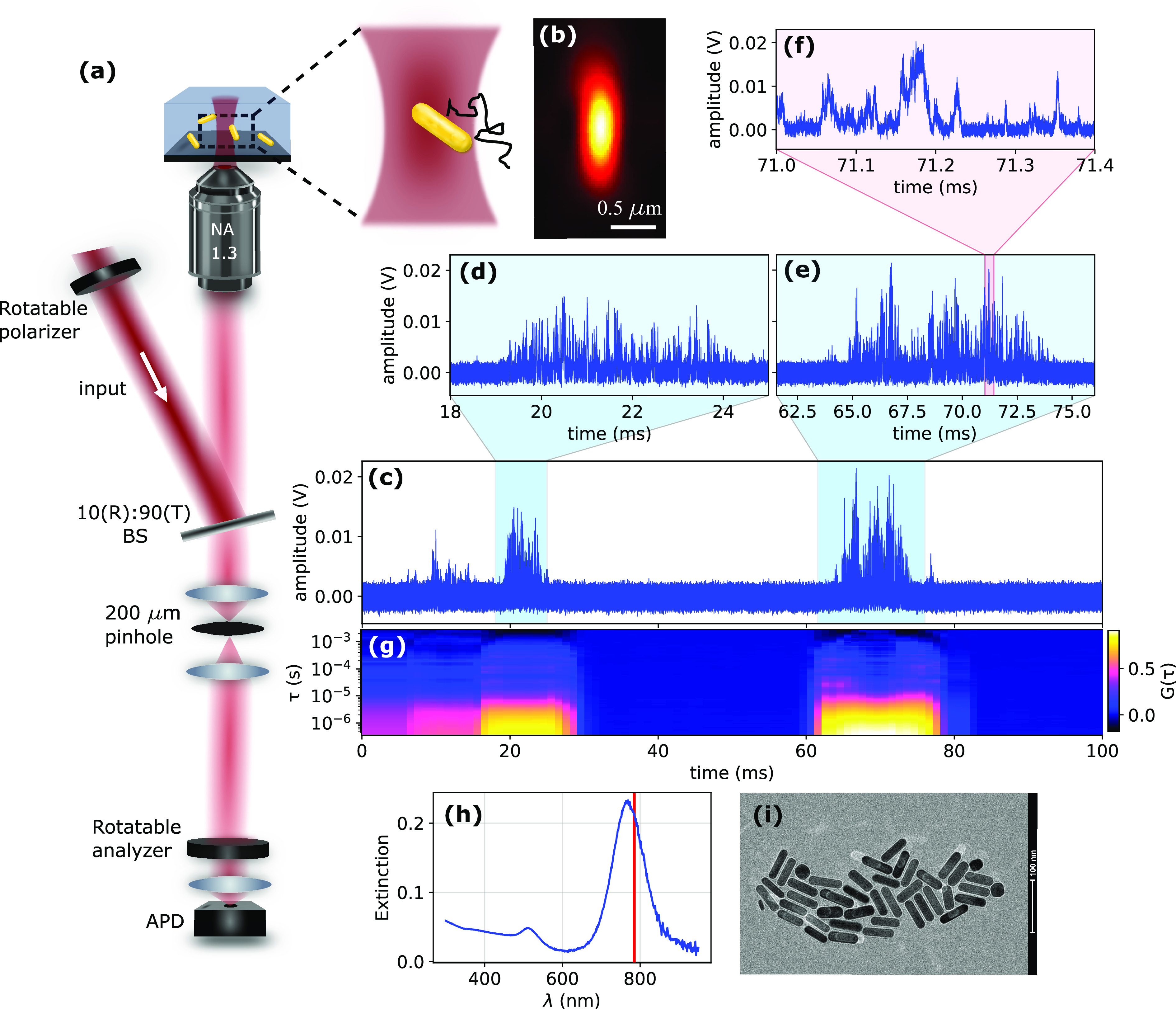
Experimental method: (a) Confocal microscope setup for detecting the high-resolution scattering time trace of freely diffusing GNRs. Linearly polarized light is reflected from a beam splitter with a small angle toward a high-NA (1.3) objective. Back-scattered light is collected through the same path and sent to an APD through a polarizing analyzer. (b) Vertical PSF. (c) Example of a scattering time trace showing two clear events due to GNRs freely diffusing into and out of the confocal volume. (d–f) Consecutive zoom-ins on highlighted events (blue, pink background), showing the sub-bursts, i.e., the fast fluctuations of the parallel-polarized intensity caused by the GNR’s tumbling motion. (g) Color-coded map of the autocorrelation function of the scattered intensity on sliding 10 ms intervals. Note the logarithmic vertical time scale. (h) Bulk extinction spectrum (blue) of the GNR solution showing a strong longitudinal plasmon peak at 768 nm. The red vertical line indicates the laser wavelength (785 nm). (i) TEM image of the used GNR sample (see section S1 in SI).

We performed our experiments using the custom-made confocal microscope (see a simplified sketch in Figure 1a) we had previously utilized for photothermal benchmarking of plasmonic biodetection assays^31^ and the fast plasmonic detection of single diffusing proteins on nanosecond timescales.^32^ In order to obtain a dark-field-like configuration, we focus the laser into a plane several micrometers away from any interface. As a consequence, the optical background noise due to scattering by impurities in the optical path is negligible in comparison to the electronic noise floor of the APD.

Individual GNRs diffusing through the confocal detection volume cause clear intensity changes (Figure 1c) with durations on the order of a few milliseconds, which we call events or bursts (Figure 1d,e). The faster intensity fluctuations within each event (Figure 1f), which we call sub-bursts, occur on timescales of few microseconds and are due to the 3D-tumbling of the GNR with respect to the incident electric field’s polarization. Since the difference in time scale between burst and sub-bursts spans almost 3 orders of magnitude, we can safely ignore translational-rotational coupling. To detect events we calculate the autocorrelation function *G*(τ) of the scattered intensity on sliding windows (Figure 1g) with a length on the order of the expected translational diffusion time of our GNRs, i.e, 10 ms. The autocorrelation function *G*(τ) is defined by:
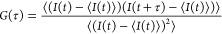
2where *I* is the scattered intensity.^33^ Intervals with *G*(τ < 50 ns) > 0.1 are recognized as events for further analysis. At the highest used GNR concentration, we find that events account for ≈ 10% of the entire recording time. From this value, we estimate that about 10% of events are potentially caused by simultaneous detection of two or more GNRs (see Supporting Information (SI) section S2). In these few cases, the measured rotational diffusion times will represent the multiparticle average and thus will have only negligible influence on our analysis.

### Rotational Correlation Function

The intense and anisotropic scattering signal from individual plasmonic GNRs can be analyzed by its autocorrelation function, as done in fluorescence or dynamic light scattering experiments on small ensembles. Upon pure rotational diffusion of a symmetric rotor such as a GNR, the rod axis performs an angular random walk that is characterized by its diffusion constant Θ. In other words, the GNR intersection with the unit sphere performs a random walk on the surface of that sphere. For a linearly polarized incident light, and neglecting the nonresonant transverse polarization of the rod, the scattered light emitted by the induced dipole is polarized along the rod’s long axis. The back-scattered field has components along the incident polarization and perpendicular to it. We call the associated signals parallel and crossed, respectively. Ignoring the numerical aperture of the collected beam, we can follow the derivation proposed by Aragon and Pecora^34,35^ for the fluorescence of a molecule. The correlation function *G*_*R*_ is given by a sum of decaying exponentials with different relaxation rates as *G*_*R*_(τ) = ∑_*l*_|*b*_*l*_|^2^e^–*l*(*l*+1)Θ τ^. Each component with angular momentum number *l* arises from the dependence of the scattered intensity on cos^*l*^ θ, θ being the polar angle of the rod axis with the incident electric field. For linearly polarized light, the scattered intensity contains degrees of *l* = 0, 2, and 4 in cos θ. Therefore, we obtain decay components at 6Θ and 20Θ only. Aragon and Pecora showed how the experimental geometry affects the measured correlation functions. They computed the weights for the *l* = 2 and *l* = 4 relaxation terms for different measurement geometries. For small detection numerical apertures, the correlation functions *G*_*R*_^||^ and *G*_*R*_^⊥^ for the parallel and crossed cases can be simplified as:^34^
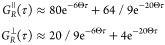
3

These expressions predict a biexponential decay of the rotational correlation, with two relaxation times in the ratio 20/6 ≈ 3.3. Due to experimental noise in real measurements, we shall not attempt to fit biexponential decays to our rotational correlation functions. Instead, we fit them with a single exponential function, which we will then identify with a single-exponential fit to Aragon and Pecora’s biexponential decay. This procedure provides us with the estimate of the rotational diffusion constant Θ (see SI section S3.1) from either the parallel (*G*_*R*_^||^) or the cross-polarized correlation function (*G*_*R*_^⊥^). We find that the latter decays are about twice as fast as the former ones. In the rest of the paper, the decay times (τ_*d*_) will be deduced from single-exponential fits, whereas the rotational diffusion constants Θ will be deduced from fits of Aragon and Pecora’s decays to the experimental correlations. Note that here, τ_*d*_ extracted from our measurements is different from the usual rotational diffusion time τ_*r*_ ≈ 1/Θ.

### Comparing Crossed and Parallel Polarizations

**Figure 2:**
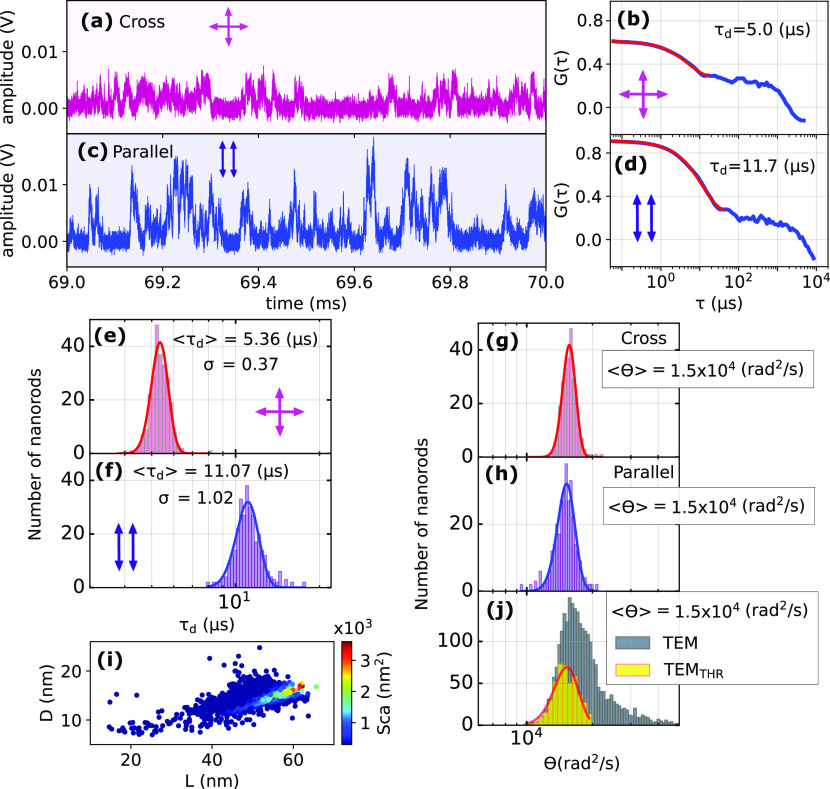
Comparison of cross- and parallel-polarized configurations: (a, c) Example sub-bursts of events recorded in cross (a) and parallel- (c) polarized configurations. (b, d) Autocorrelations (blue) of the entire events (a, c) and single-exponential fits to their fast component (red). (e, f) Histograms of single-exponential decay times extracted from 120 events for crossed and parallel polarizations and fits to Gaussian curves (solid lines) with mean values ⟨τ_*d*_⟩ and standard deviations σ. (g, h) Histograms of rotational diffusion coefficients Θ determined from decay times (e, f) according to Aragon and Pecora’s analysis. (i) Distribution of length and diameter of GNRs extracted from TEM with calculated color-coded scattering cross section σ_*sca*_ for each nanorod at 785 nm. The color scale indicates the scattering cross section at that wavelength (see Figure S10 in SI). (j) Histograms of Θ-values calculated from TEM data. The gray (TEM) histogram contains all rods, and the yellow one is for the subset of resonant rods with sufficiently large σ_*scat*_ (see SI section S4). Solid lines in (g, h, j) are fits to Gaussian distributions.

To obtain experimental data that allow for a direct comparison with eq 3, we performed measurements of GNR diffusion with the incident and analyzed polarizations in crossed and parallel configurations. Excerpts of time traces obtained for both configurations are depicted in Figure 2a (cross) and Figure 2c (parallel). The maximum scattered intensities obtainable for the same GNR in parallel and crossed configurations exhibit a ratio of *I*_*parallel*_/*I*_*crossed*_ = 4. As a consequence, we find overall higher intensities, and therefore better signal-to-noise ratios (SNRs), using the parallel-polarization configuration.

Moreover, we find that sub-bursts recorded in cross-polarized configuration occur on faster timescales than their parallel counterparts as is evident from the autocorrelation decay times τ_*d*_ shown in Figure 2b,d. A statistical analysis of τ_*d*_ values determined from more than 100 events obtained in either configuration (Figure 2e,f) reveals a ratio of τ_*d*,||_/τ_*d*,⊥_ = 2.06. This closely matches the theoretically expected value of 2.0, which can be obtained by fitting a single exponential to Pecora’s functions (eq 3); see SI Figure S5 for simulation and Figure S6 for theory. The prefactor of the e^20Θ^ term in Pecora’s *G*_||_ formula (equation. 3) is significantly smaller than the one for the e^6Θ^, hence we have approximated the diffusional rotation constant Θ for parallel polarized configuration as . We further find that the Θ-distributions determined from the decay times found for both polarization configurations exhibit excellent mutual agreement (Figure 2g,h). In order to compare the experimentally obtained Θ-distributions (Figure 2g,h) with the distribution expected for the size distribution of our GNR sample, we first recorded high-resolution TEM images (see Figure 1i and SI section S1). From these, we then determined length and diameters of >2500 individual GNRs (average length: 51.7 nm, average diameter: 14.9 nm), excluding spherical particles. Next, we calculated the rotational diffusion coefficients for each GNR via eq 1, considering a 2.2nm CTAB layer, to obtain the expected Θ_*TEM*_-distribution (Figure 2j, gray). Surprisingly, this distribution is significantly broader than those obtained from our optical measurements (Figure 2g,h). We assign this difference to the selection bias created by optical resonance. Only nanorods with favorable aspect ratios, i.e., LSPRs in resonance with the probe laser, possess sufficiently large scattering cross sections to enable detection, resulting in the selection of a GNR subpopulation. This narrowing of the diffusion constant histogram by the plasmon resonance has been noted earlier by Yuan et al.^36^ This effect appears surprising at first sight, as the plasmon resonance is essentially a function of the rod’s aspect ratio and does not seem to be an efficient way to select rods on the basis of their hydrodynamic volume. Yet, applying a threshold determined by our experimental SNR for the scattering cross sections calculated for each nanorod in our TEM data set (see Figure 2i and SI section S4), we indeed obtain a narrower distribution of rotational diffusion constants (Figure 2j, yellow) with a shifted peak position. We assign the narrowing effect to the, in comparison to the aspect ratio distribution, narrower distribution of nanorod diameters, and the peakshift to the detuning between our laser’s wavelength (785 nm) and the GNR’s average LSPR (Figure 1h). Let us stress here that the selection bias is a pure plasmonic effect and does not depend on the solvent’s viscosity nor on variations of the hydrodynamic volume of the diffuser due to binding of molecules to the GNR surface. Therefore, this subpopulation can be used to monitor changes of viscosity or of hydrodynamic volume of the GNRs (see section S5 in SI). We also confirmed that a small shift of the plasmon resonance upon adsorption of analytes or upon changes of the refractive index of the surrounding medium will have a negligible effect on the selected subpopulation and therefore on the determined rotational decay times (see section S5 of the SI).

Further, we find that the Θ-distribution obtained in the cross-polarized configuration (Figure 2g) is narrower than its parallel configuration counterpart (Figure 2h). This additional narrowing could arise from two effects: (I) The stronger selection of events is due to the higher signal required for detection in the crossed configuration (SI section S4) and (II) the number of cross-polarized sub-bursts within each event is higher in comparison to its parallel-polarized counterpart (SI section 3.3), yielding better statistics. In our configuration, effect (I) is significantly stronger. The excellent match between the Θ-distributions obtained via optical and TEM measurements, especially regarding the distribution means, confirms the accuracy and reliability of our optical rotational diffusion measurements.

In summary, the parallel-polarized configuration provides a better SNR, while it also maintains a sufficiently large number of sub-bursts per event for the accurate determination of rotational diffusion constants. Therefore, we will use it throughout the following parts of this manuscript without further indication. However, we want to note that for GNR samples with different dimensions, in particular larger NRs, the lower ratio between sub-burst and event durations as well as the overall higher scattering cross section can make the crossed configuration the more favorable choice.

### Rotational Hot Brownian Motion

**Figure 3:**
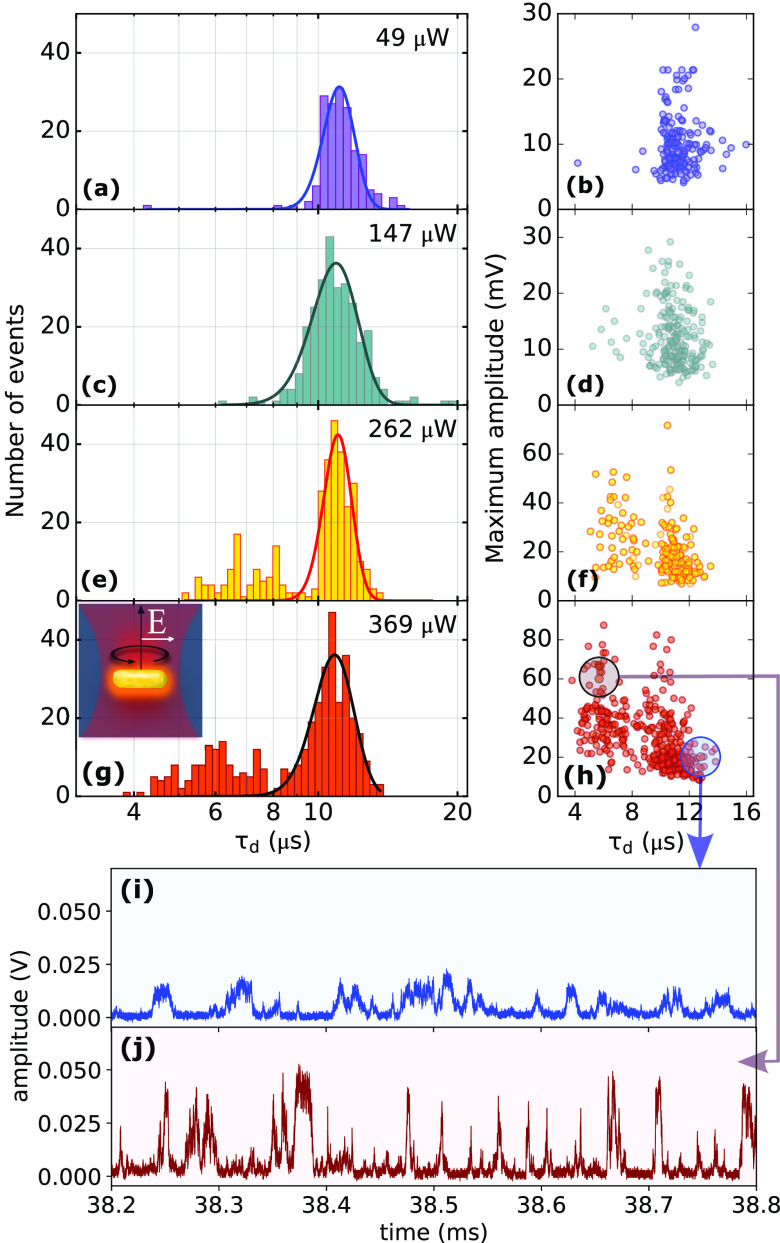
Power dependence of tumbling times: (a, c, e, g) Histograms of tumbling times obtained at the indicated laser powers. Solid lines are fits by Gaussian curves. (b, d, f, h) Scatter plots of tumbling times versus maximum amplitudes from individual GNR events obtained at the powers indicated in the histograms to their left. (i, j) Representative excerpts of time traces (full traces: SI Figures S15 and S16) of two events highlighted in (h) showing the qualitative differences between the two populations (i, main population; j, fast population).

All of the previous measurements were done with the fixed power 49 μW in the focal plane of the objective. We now deviate from this setting to investigate the effect of the laser power on the rotational diffusion. For this we characterize each event by the decay time τ_*d*_ of its correlation function and by the maximum amplitude of the event’s highest sub-burst, which we take as a proxy for the scattering cross section of the particular nanorod causing that burst. We find that with increasing power, a growing population of bursts characterized by significantly shorter (τ_*d*_ = 4–8 μs) rotational decay times appears (Figure 3a,c,e,g). This second faster population also exhibits maximum amplitudes that grow significantly with increasing power (Figure 3b,d,f,h).

The qualitative difference between the two subpopulations is well represented by the example event excerpts shown in Figure 3i,j, showing sub-bursts with comparatively low amplitudes and slow dynamics occurring at slower rate (Figure 3i) and sub-bursts with comparatively high amplitudes and fast dynamics occurring at a higher rate (Figure 3j), respectively. To gain insight into the second population’s origin we have to consider that, together with increasing power, not only the SNR increases, but also the influence of photothermal effects (I) and optical forces (II) on the GNRs tumbling motion increase: (I) Photothermal heating of plasmonic particles^37^ creates a temperature gradient around the particle, which travels with the particle as heat diffuses much faster (thermal diffusivity of water: 1.46 × 10^–5^ m^2^/s)^38^ than the translational diffusion of the particle itself (*D* ≈ 3 × 10^–11^ m^2^/s). This temperature gradient and the associated viscosity gradient cause the particle to diffuse faster than a nonilluminated particle. The effect of the heating on rotational diffusion is called rotational hot Brownian motion and was investigated theoretically by Rings et al.^26,27,39^ for isotropic light absorption. Our situation here is more complex, as the heating power depends on the tumbling motion itself. Furthermore, we note that rotational hot Brownian motion has a higher effective temperature and lower effective viscosity than translational hot Brownian motion,^25^ because rotational velocity fields decay faster with distance to the particle than translational velocity fields.^26^ We expect rotational hot Brownian motion to be much more active for a big resonant rod (Figure 3j) than for smaller or nonresonant ones (Figure 3i). Indeed, Figure 3i,j, recorded with a temporal resolution well below 500 ns (see SI Figure S15g), qualitatively confirms an excess of short events in the pink trace compared to the blue one. Simulations of rotational diffusion rates under heating are presented in the SI (section S6.1) and confirm the appearance of the second population due to rotational hot Brownian motion.

(II) A further possible complication at high laser intensities is the optical torque which tends to align the nanorod’s axis with the laser polarization for positive polarizability when its plasmon resonance lies at a shorter wavelength than the laser, and to disalign it for negative polarizability, when the resonance is at longer wavelengths than the laser.^25,40^ At the highest power in our measurements, (369 μW), the maximum optical potential acting on a nanorod with the average size in our sample is about 1.4*k*_B_*T* (see SI section S7). Optical (dis-)alignment effects are therefore not negligible in the above histograms. Similarly, optical trapping effects and radiation pressure effects due to photon absorption, in addition to complex thermophoretic effects may play a significant role in the duration of translational events at the maximum power.

Photothermal effects and the influence of optical forces will both increase with particle size and proximity of the laser’s wavelength to the GNR’s LSPR. Thus, these effects are strongest for a subpopulation of GNRs with both larger-than-average diameters and smaller-than-average LSPR-laser detuning. This subpopulation then experiences faster rotational diffusion. Furthermore, this population will grow in size as the power is increased.

In the rest of this work, we keep the laser power at 49 μW, so that all effects due to heating and associated optical forces remain negligible.

### Rotational Diffusion in Dilute PVA Solutions

Being sensitive to the hydrodynamic volume of a nanoparticle, rotational diffusion can be used to directly detect binding of (bio)molecules to the nanorods. This method of detection offers several advantages: (I) GNRs can be used as sensors in suspended form i.e. without the need for surface immobilization required for typical plasmonic assays,^41,42^ broadening the range of possible applications significantly. (II) Rotational diffusion is orders of magnitudes faster and more sensitive to size variation (Θ ∝ *L*^–3^) than translational diffusion (*D* ∝ *L*^–1^).^30,43^ Assays based on observation of rotational diffusion thus promise superior speed and sensitivity in comparison to translational-diffusion-based assays.^44^ (III) Measurements of rotational diffusion times are, in contrast to measurements of translational diffusion times,^33,45−47^ intrinsically independent of the size and shape of the confocal volume. This makes it significantly easier to introduce standards and to directly compare results from measurements performed in different geometries or indices of refraction.

To demonstrate this idea, we studied the rotational diffusion of GNRs in the presence of very low concentrations (tens of ppb to 100 ppm) of poly(vinylalcohol) (PVA) in water. We consider this system as a model for the unspecific detection of proteins such as Bovine Serum Albumin (BSA).^30^ An example of BSA binding detected by GNR rotational diffusion is presented in section S9 of SI. A similar detection scheme could apply to biomolecules binding to suitable receptors covalently attached to the GNRs.

The PVA chains we use in this work contain on average 2800 monomers and possess an average molecular mass of 125,000 g/mol. They are also significantly (88%) hydrolyzed and thus will not cross-link within coils while suspended in water.^48^ We use very low concentrations of PVA (up to 100 ppm in mass), and hence, we work in the dilute regime,^49^ where according to Flory’s model, polymer self-avoiding random coils are well separated from each other in the solution.^50^ In consequence, the relative change of viscosity (on the order of 10^–3^) is negligible^51^ and will have no influence on the measured rotational diffusion of GNRs.

**Figure 4:**
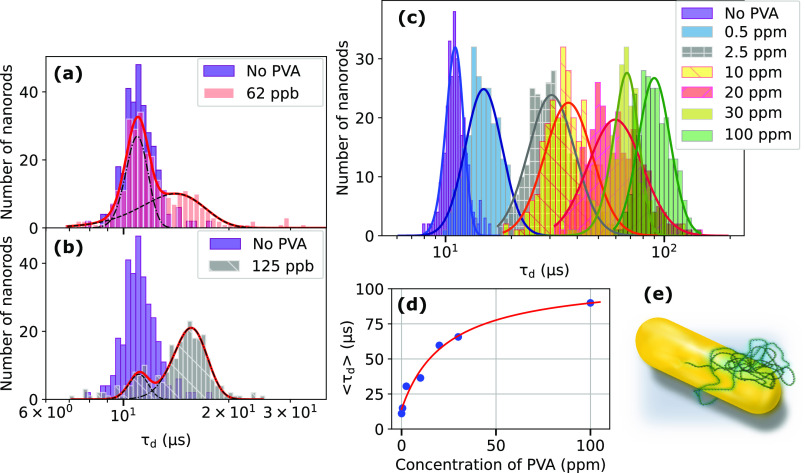
Dependence of GNR rotational diffusion on the PVA concentration: Purple histograms in (a–c) represent the GNR tumbling times in water for reference. (a) Histogram of GNR tumbling times for 62 ppb PVA in water (pink). (b) Histogram of tumbling times for 120 ppb PVA in water (gray). Solid lines (a, b) are fits with double (red) and single (black) Gaussian curves. (c) Histograms of GNR tumbling times for PVA in water concentrations up to 100 ppm. Each histogram comprises 220 events. Solid lines are fits to log-normal curves, which show narrowing by approaching the saturated state. (d) Mean value of the tumbling time distributions from (c) plotted versus the corresponding PVA concentration (dots). Solid line: Fit to a Langmuir isotherm, corresponding to an affinity of *K* ≈ 20 ppm. (e) Illustration of a PVA random coil bound to a GNR.

We start our measurement series (parallel polarization configuration, power 49 μW) with very low concentrations of PVA, ensuring that the number of polymer coils per unit volume is lower than the number of GNRs. The results of these measurements are displayed in Figure 4a,b, whereas a histogram of rotational times of nanorods in pure water is presented in purple and is the same as those in Figure 2f and Figure 3a. It will serve as a reference for the detection of PVA binding. At the lowest PVA concentration, 62 ppb, we have 0.5 polymer coils per nanorod in the solution ensuring an excess of PVA-free GNRs. Indeed, the pink histogram of tumbling times in Figure 4a clearly shows two populations of GNRs. The first one overlaps perfectly with the purple reference histogram. We assigned this population to free GNRs. The second population displays slightly but significantly longer tumbling times, which we assign to an increase in hydrodynamic volume by about 30%, caused by binding of one PVA coil to some nanorods (see scheme in Figure 4e).

Upon doubling the PVA concentration to 125 ppb (≈1 coil per GNR), the second population (gray histogram of Figure 4b) grows considerably and shifts to longer tumbling times suggesting a further increase in hydrodynamic volume due to the binding of more polymer coils to the GNRs. Note the small remaining population of free rods. The larger width of the second population both in (a) and (b) compared to that of the free rods (purple) suggests variations of the size and number of the polymer coils bound to the rods, and heterogeneity of their binding sites. The result of this experiment is notable: Although the distribution of GNR volumes is rather broad, plasmonic selection narrows the histogram sufficiently to detect a fairly small variation in hydrodynamic volume by only about 30%.

We then continue our measurement series by gradually increasing the PVA concentration to 100 ppm (Figure 4c). The first τ_*d*_ histogram in Figure 4c (blue) appears monomodal (a single population), as it is considerably broader than those of the very dilute cases. We assign this width to fluctuations in the number and sizes of the bound PVAs. A further increase of the PVA concentration, in Figure 4c, clearly causes a further shift of the histograms until, at around 100 ppm (the green histogram in Figure 4c), the histogram stops shifting and starts narrowing. We interpret this observation as an indication that most of the binding sites on the GNRs are now occupied by PVA. All of the histograms in Figure 4c are well fitted with log-normal distributions. By plotting the mean tumbling time as a function of PVA concentration (see Figure 4d), we find a clear saturation behavior for the hydrodynamic volume *V*_*H*_ which can be well modeled by a Langmuir isotherm:
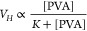
4where the binding affinity *K* = *d*/*a* is the ratio of the dissociation (*d*) and association (*a*) rates of adsorbents on the available sites at the GNR surface. We assume the hydrodynamic volume scales linearly with the number of polymer molecules adsorbed. With the values of Figure 4d, we find a limiting hydrodynamic volume of about 6 times the initial volume of the nanorod, corresponding to about 5 average PVA coils adsorbed at the GNR surface.

It is interesting to note that the initial adsorption sites shown in Figure 4a,b measured at very low concentration correspond to a much larger adsorption probability than the sites occupied at the later stages of the adsorption reaction (see SI section S8). We assign this difference to heterogeneity in the binding constants of the different sites at the GNR surface. The high affinity of the initially occupied sites allows us to detect even very weak concentrations of PVA with high confidence, as would specific receptors for the detection of a low concentration of biomolecules.

## Conclusion

We studied the rotational diffusion of individual GNRs on a burst-by-burst basis. Large numbers of events provide a good statistical significance. Surprisingly, the selection of resonant plasmonic nanorods produces comparatively narrow histograms of tumbling times, which are sensitive to changes in temperature, viscosity, and hydrodynamic volume of the particles. Rotational diffusion constants can be extracted from crossed- or parallel-polarization measurements, which provide consistent results in good agreement with theory and dimensions of the nanorods measured by TEM. As an example of a local change of temperature and viscosity of the solvent, we investigated the effect of laser power on the rotational diffusion of the GNRs. The effect of heating is moderate at low power but becomes more and more pronounced at high powers as a shortening of the tumbling times of the largest and/or most resonant rods, which also are the hottest ones. The interpretation of these data, however, is complicated by possible effects of optical forces and torques, which may align or disalign the nanorods with respect to the incident polarization depending on their plasmon resonance. A second use of rotational diffusion is as a sensor of ligand binding down to very low concentrations. We have investigated tumbling time histograms in the presence of a very low concentration of PVA, which binds to GNRs. We find a saturation of adsorption at about 100 ppm, the process being well described by a Langmuir isotherm. At PVA concentrations lower than 100 ppb, however, the affinity for adsorption is much greater, which points to heterogeneity in the adsorption sites for this polymer. These observations allow the possibility to use tumbling nanorods as probes for low concentrations of ligands, provided specific receptors can be attached covalently to their surface. Another intriguing application of our burst-by-burst measurements of rotational diffusion would be the study of heterogeneous environments, such as porous materials or live cells. Detailed studies of rotationally diffusing plasmonic probes reveal specific adsorption sites and local mobilities with high resolution in time and space.

## Methods

### Optical Setup and Electronics

The probe beam (785nm wavelength) is sent to the objective over a plate beam-splitter inclined at a small angle (7°), in order to minimize changes to the polarization states of both the incident and the collected light. The linear polarization of incident and collected scattered light can be set and analyzed arbitrarily via the rotation of a λ/2-plate and a Glan-Thompson polarizer. The intensity of the collected scattered light is measured via a fast avalanche photodiode (APD, max. bandwidth 400 MHz) and digitized at a 50 MHz sampling rate using a fast oscilloscope (LeCroy wavesurfer 200 MHz) after a low-pass filter (SI190 MHz) We determined a confocal volume of 0.35 fL from the point-spread function (PSF) obtained from a 3D-scan over a GNR (Figure 1b).

### Sample Preparation

#### Water Suspensions

The suspension of CTAB-coated GNRs was purchased from Nanopartz (A12-10-780-CTAB-DIH-1–25). Circular cover glasses with thickness number 1 and 25 mm diameter were sonicated in acetone for 30 min and ethanol for 30 min and rinsed with milli-Q water. Then they were mounted in a flow cell in a confocal microscope setup.

#### PVA Solutions

Poly(vinyl alcohol), 88% hydrolyzed with average molecular weight of 125,000 g/mol, was purchased from Janssen Chemica. To obtain low concentrations of PVA, we first prepared solutions of 0.01% PVA in milli-Q water by gentle stirring (300 rpm) at 40 °C for 4 h. Afterward, we let the solution cool to room temperature. Then we sonicated the GNR solution for 5 min and prepared a mixture of PVA solution and milli-Q water to achieve the desired concentration of PVA while maintaining a fixed concentration of GNRs (4.0× 10^11^ particles/mL). PVA- and GNR-containing solutions are then mixed right before the start of the experiment.

## Supporting Information Available

Additional data and figures (ZIP)
TEM; estimation of events overlap; simulations of rotational diffusion; LSPR-based detection; the rotational diffusion times are independent on laser detuning from LSPR; rotational Hot Brownian motion; optical torque; rotational diffusion in PVA solutions; observation of protein adsorption (PDF)
