# Supplementary material for: Burst-by-Burst Measurement of Rotational Diffusion at Nanosecond Resolution Reveals Hot-Brownian Motion and Single-Chain Binding
Source: ACS Nano. 2023 Jun 23;17(13):12684–92. doi: 10.1021/acsnano.3c03392 (PMC10339794; doi:10.1021/acsnano.3c03392)
Supplement: Supplementary file 1 — nn3c03392_si_001.zip [file nn3c03392_si_001.zip › S_BSA.pdf]

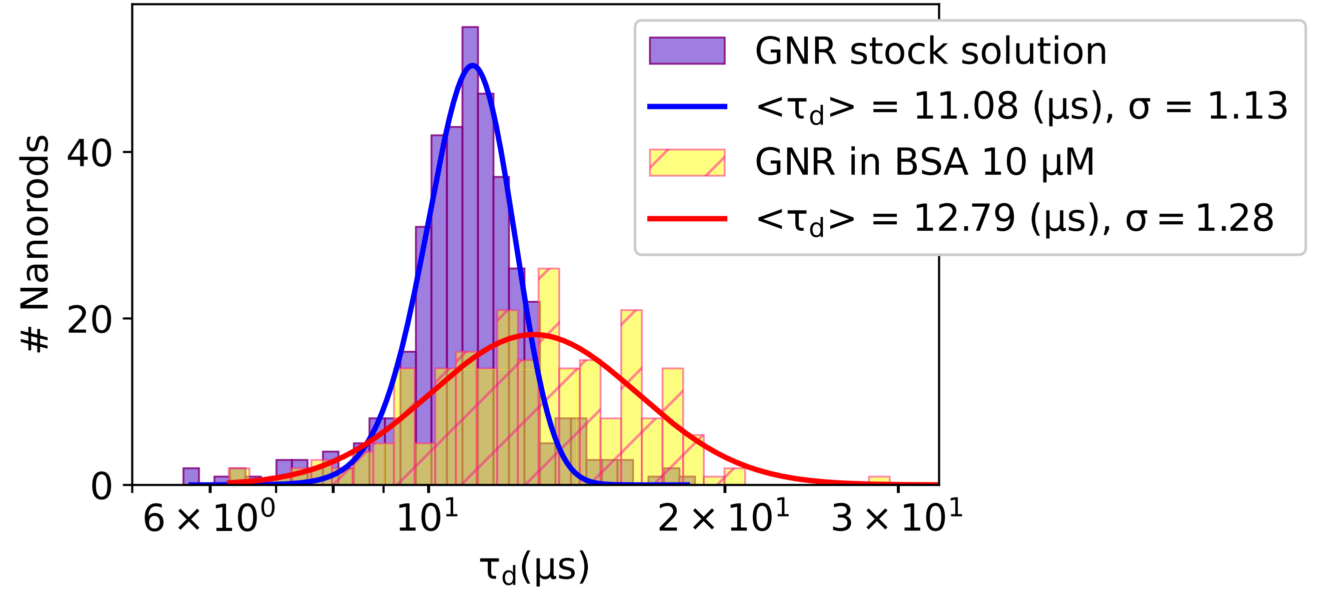

GNR stock solution
<τ_d> = 11.08 (μs), σ = 1.13
GNR in BSA 10 μM
<τ_d> = 12.79 (μs), σ = 1.28
Nanorods
0
20
40
6 × 10^0
10^1
2 × 10^1
3 × 10^1
τ_d(μs)
