# Supplementary figures and images for: Burst-by-Burst Measurement of Rotational Diffusion at Nanosecond Resolution Reveals Hot-Brownian Motion and Single-Chain Binding
Source: ACS Nano. 2023 Jun 23;17(13):12684–92. doi: 10.1021/acsnano.3c03392 (PMC10339794; doi:10.1021/acsnano.3c03392)

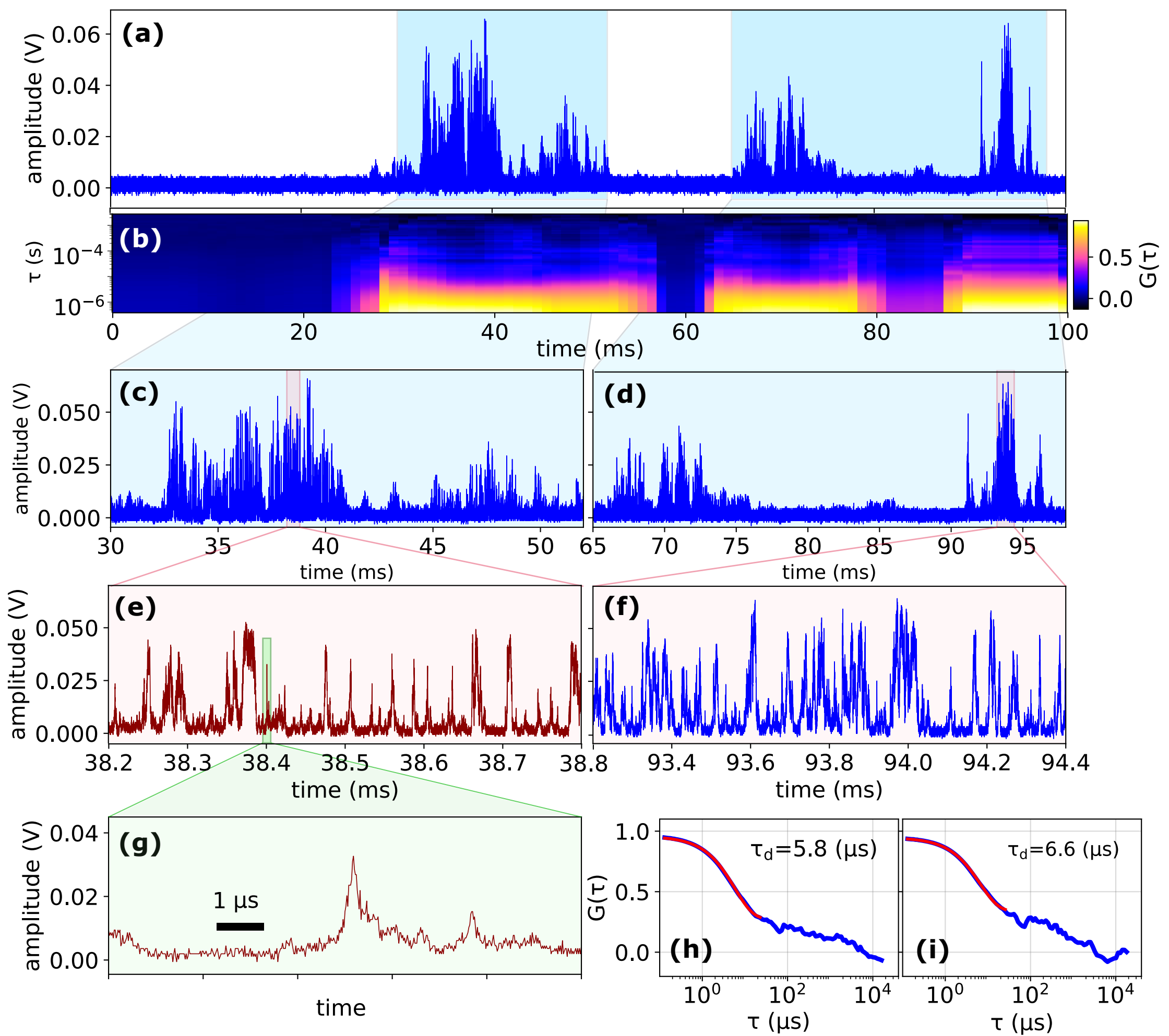

Supplement: Supplementary file 1 — nn3c03392_si_001.zip [file nn3c03392_si_001.zip › S_HBM trace.pdf]

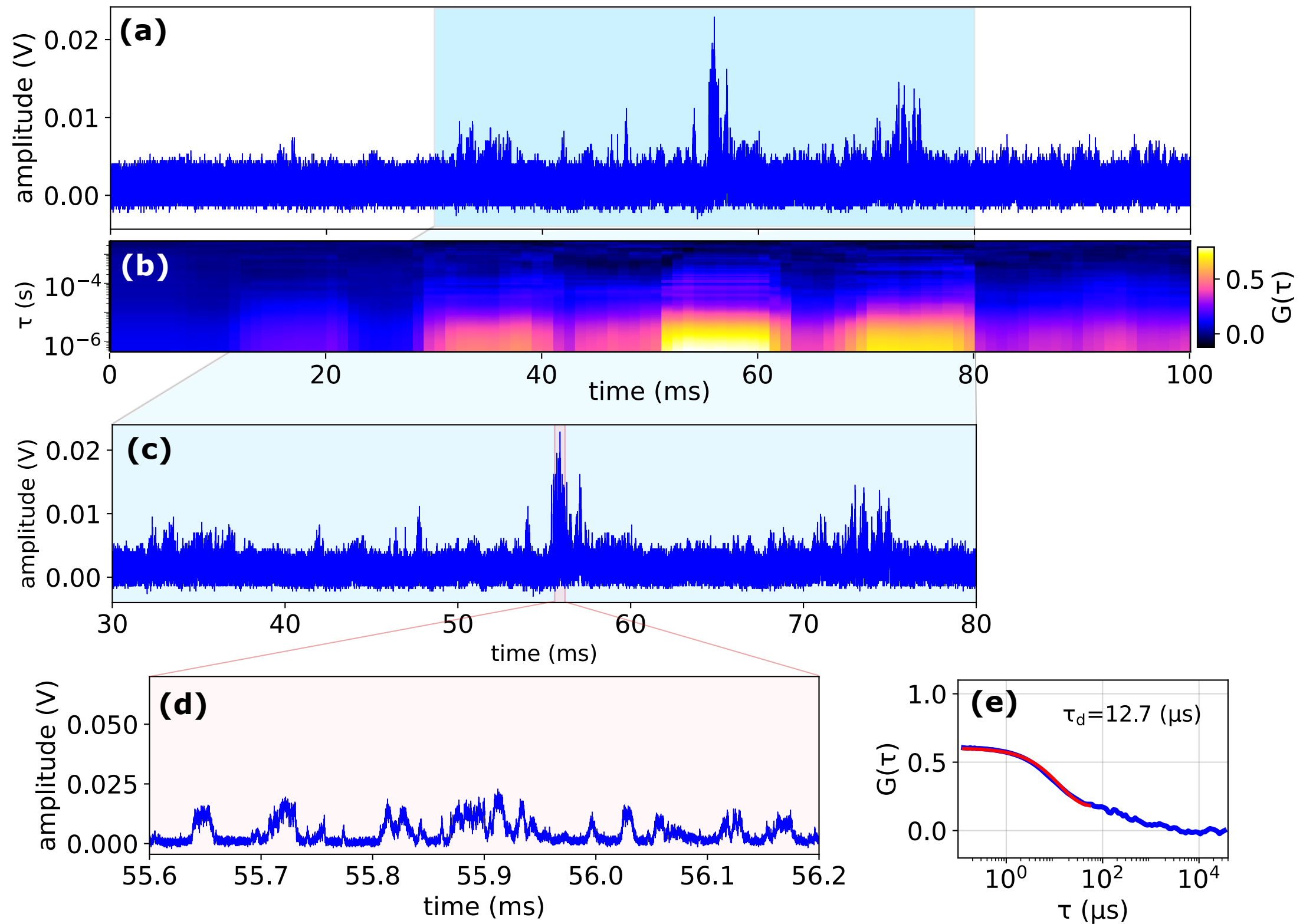

Supplement: Supplementary file 1 — nn3c03392_si_001.zip [file nn3c03392_si_001.zip › S_HBM_slow.pdf]

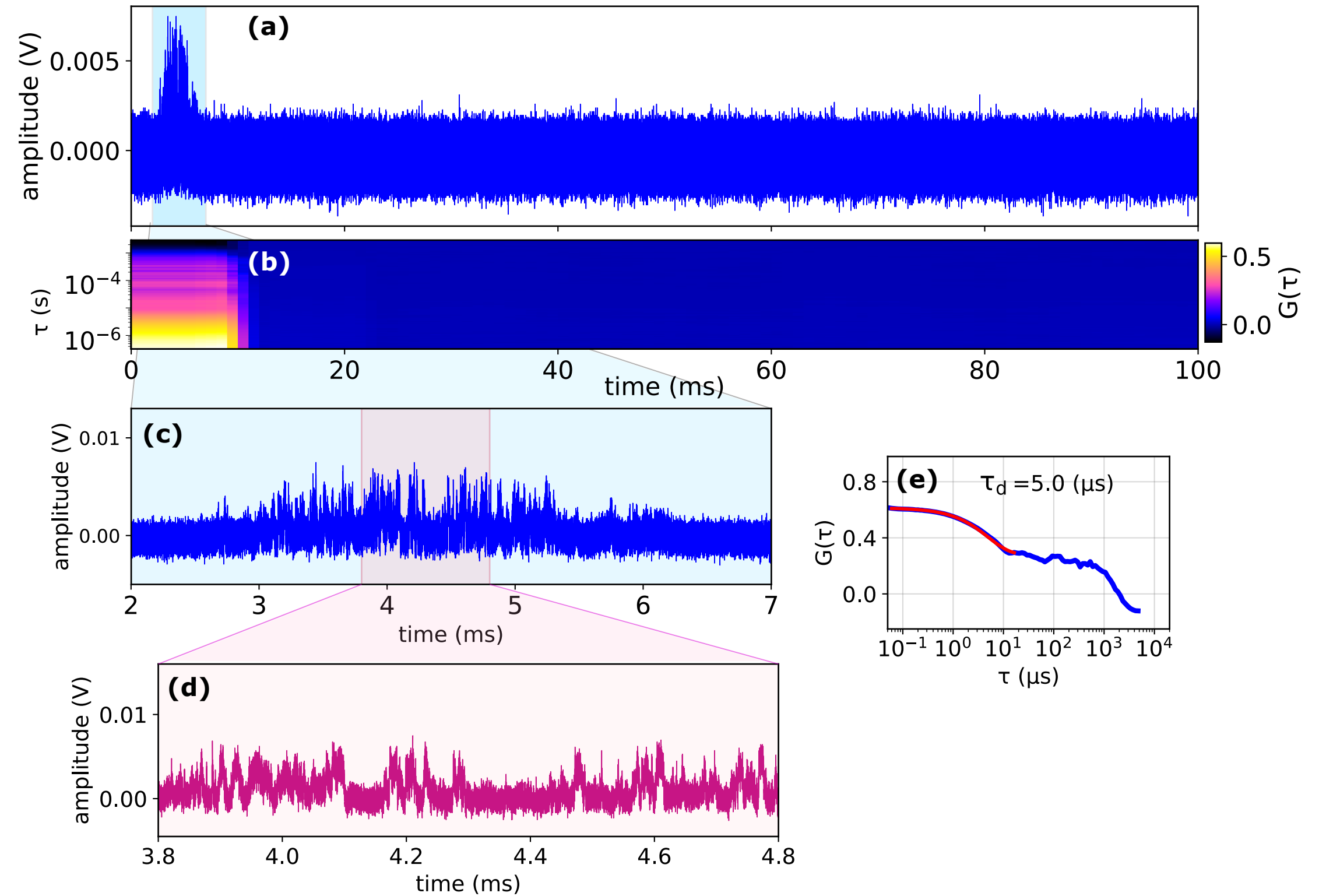

Supplement: Supplementary file 1 — nn3c03392_si_001.zip [file nn3c03392_si_001.zip › S_cross_trace.pdf]

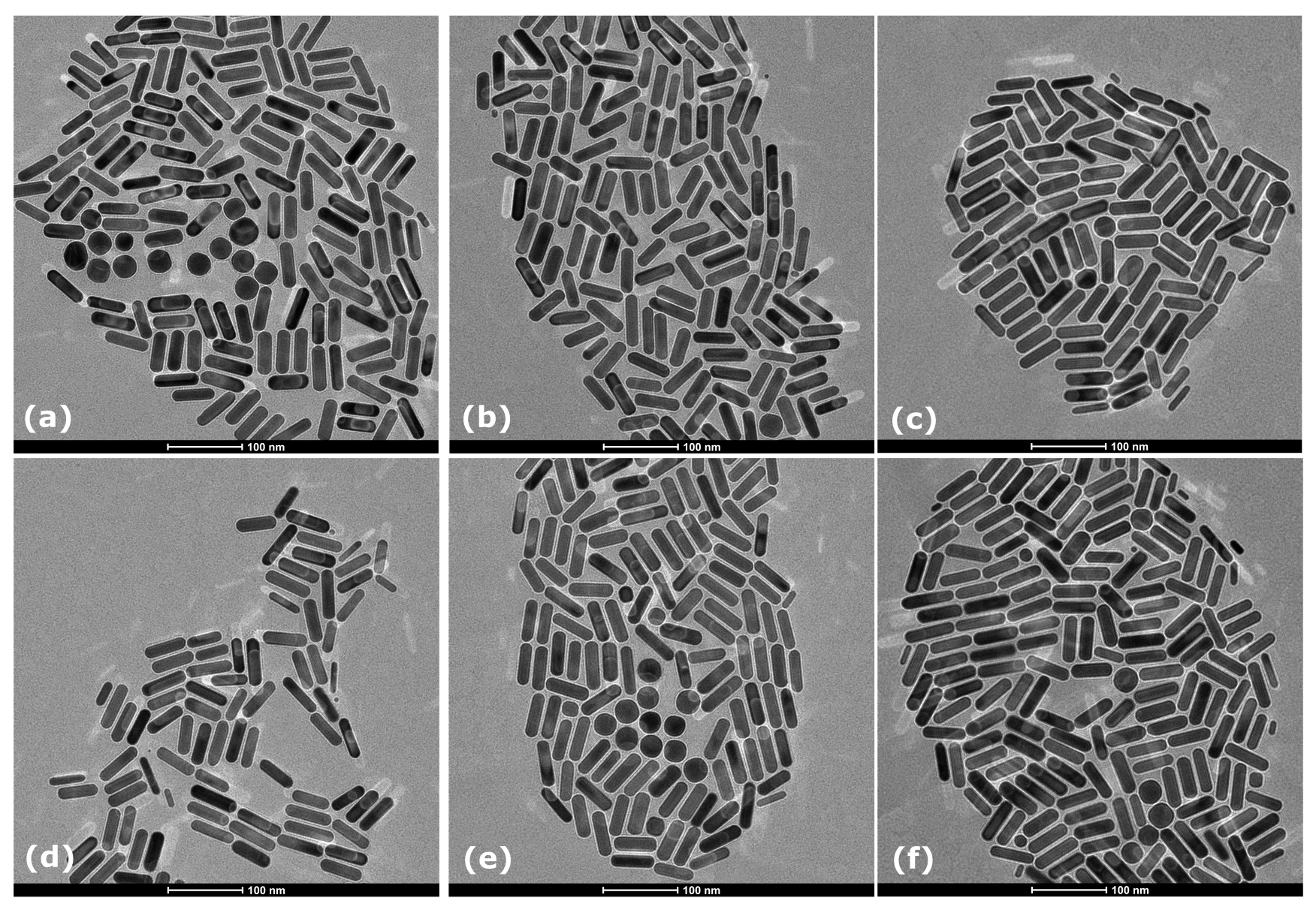

Supplement: Supplementary file 1 — nn3c03392_si_001.zip [file nn3c03392_si_001.zip › S_TEM.png]

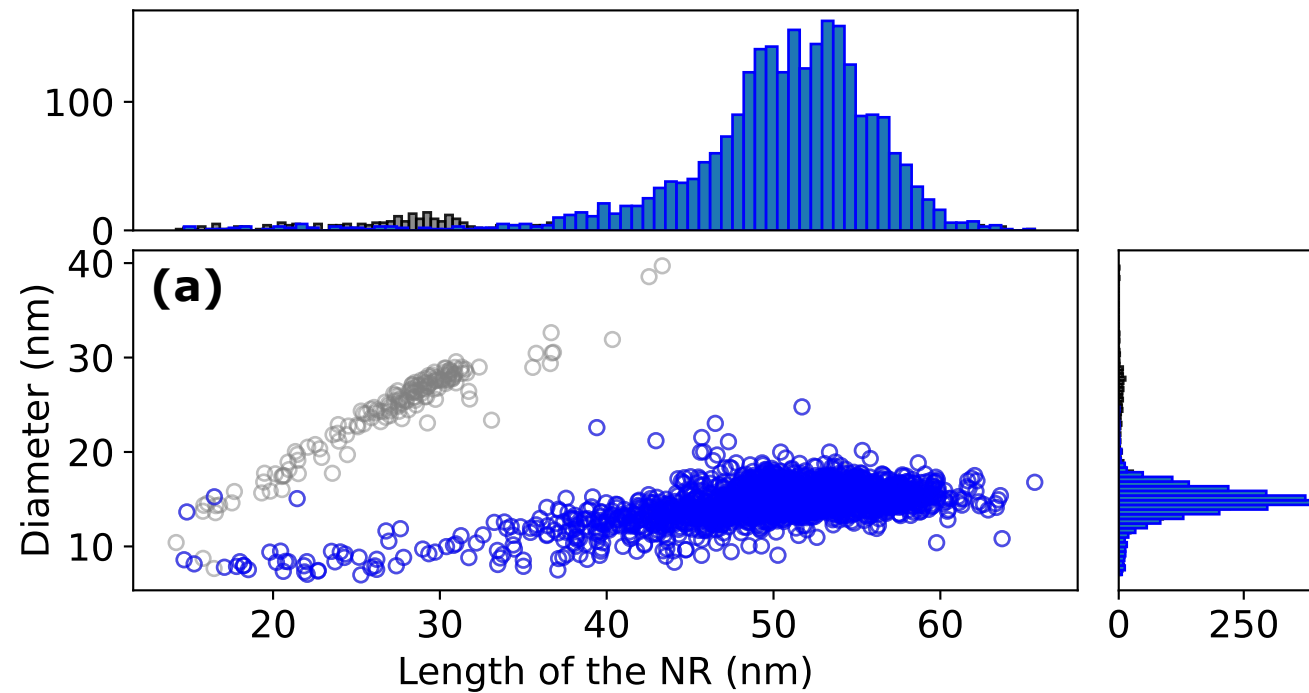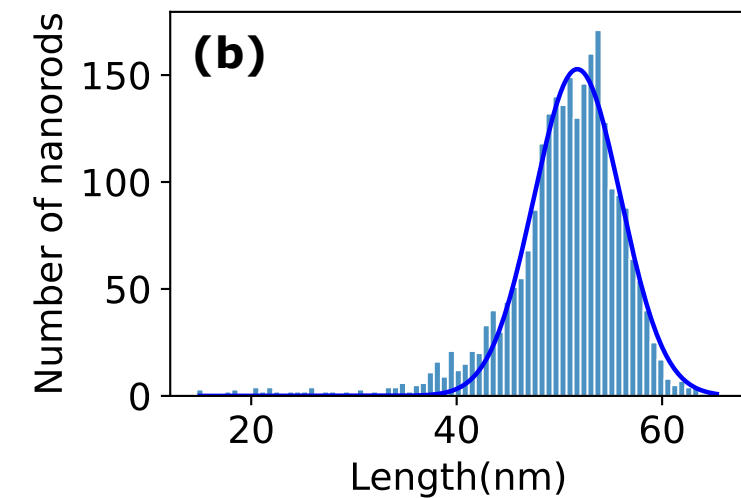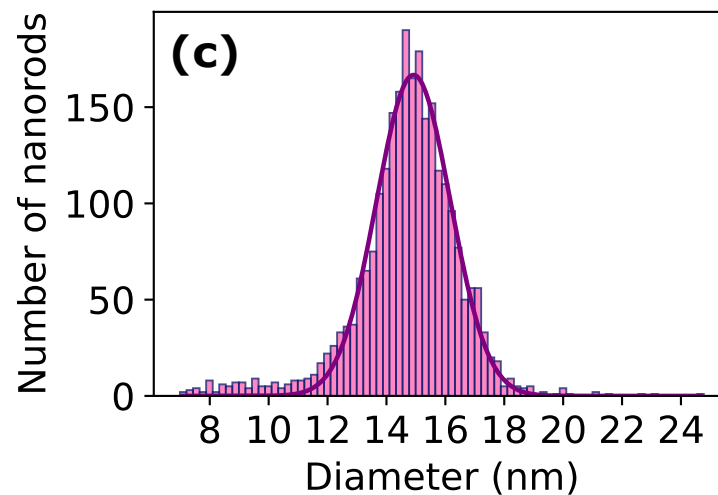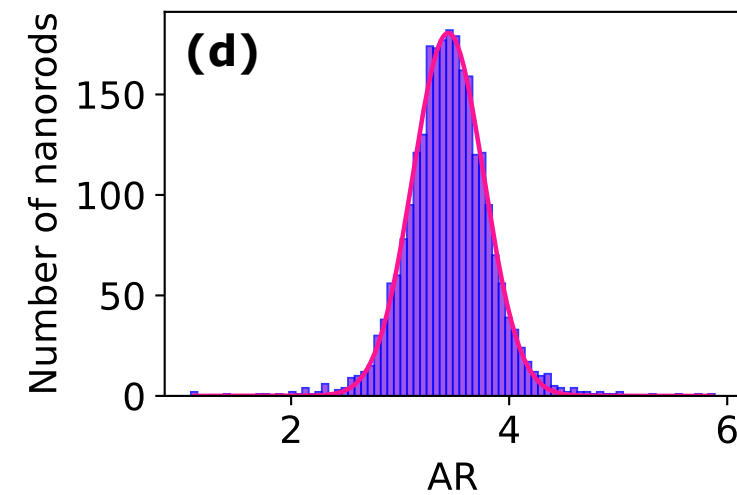

Supplement: Supplementary file 1 — nn3c03392_si_001.zip [file nn3c03392_si_001.zip › S_TEM_hist.pdf]

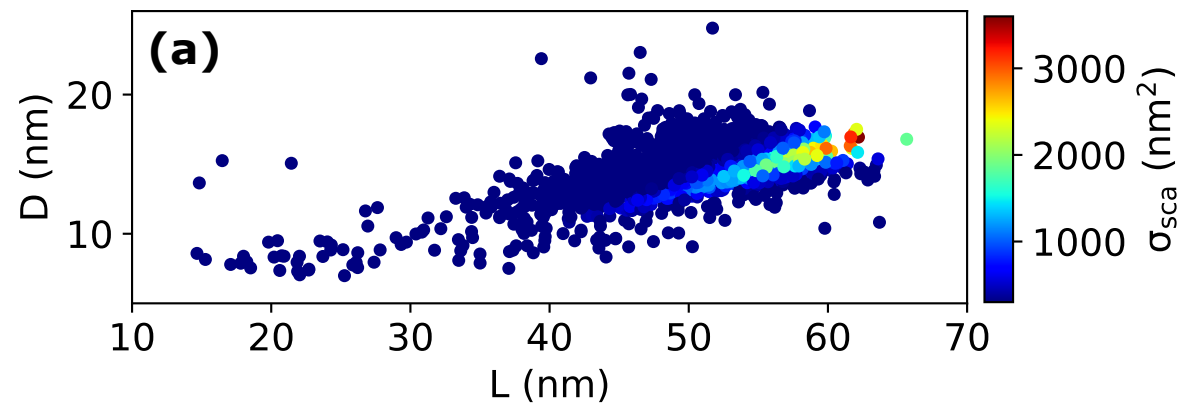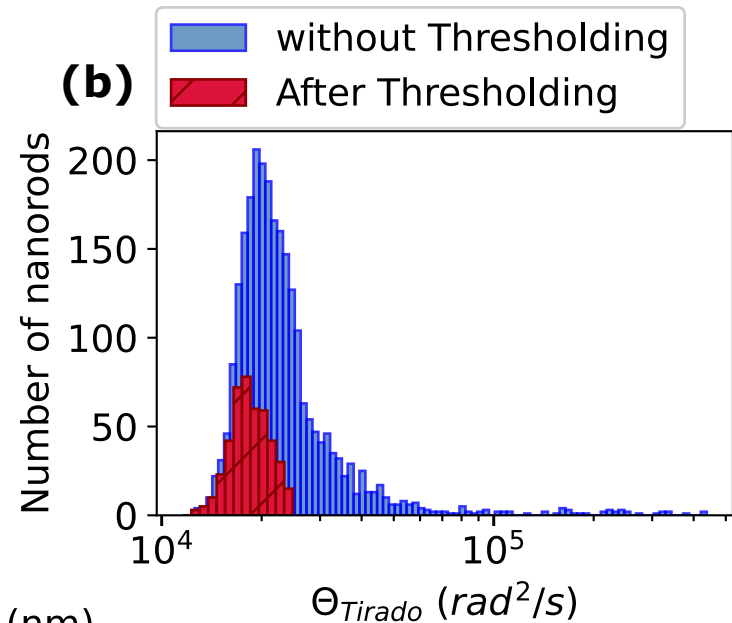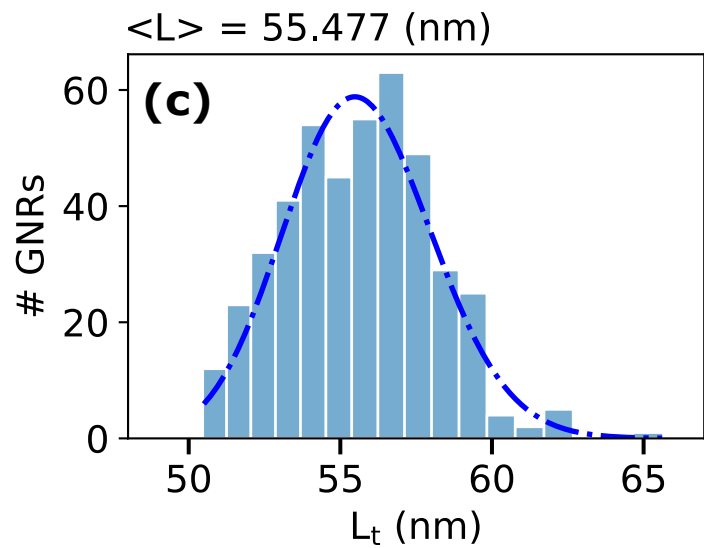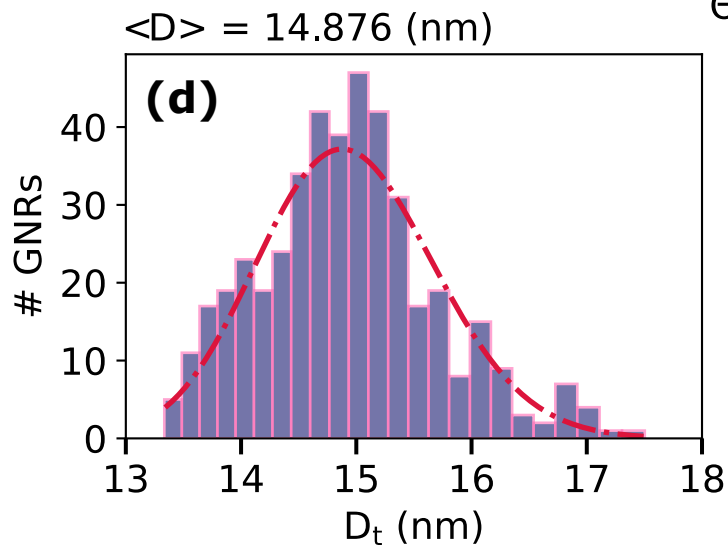

Supplement: Supplementary file 1 — nn3c03392_si_001.zip [file nn3c03392_si_001.zip › S_TEM_scat.pdf]

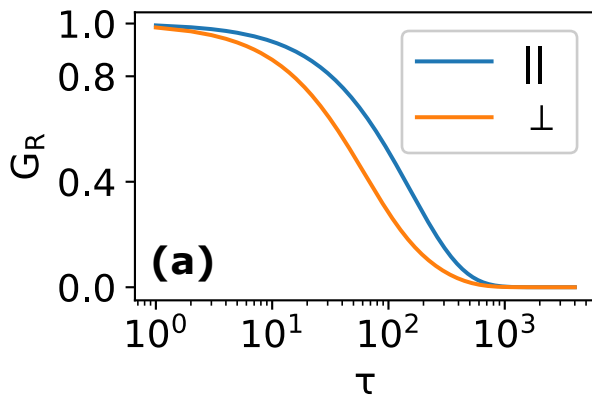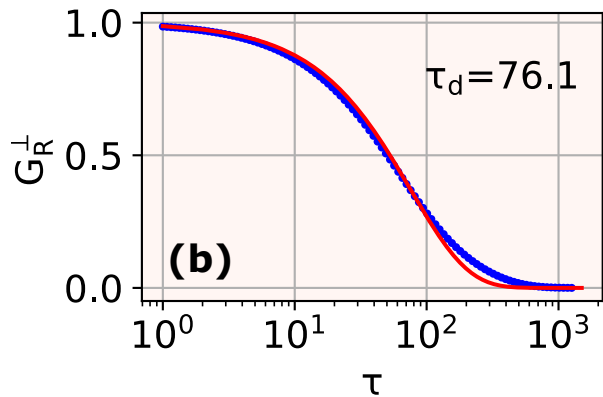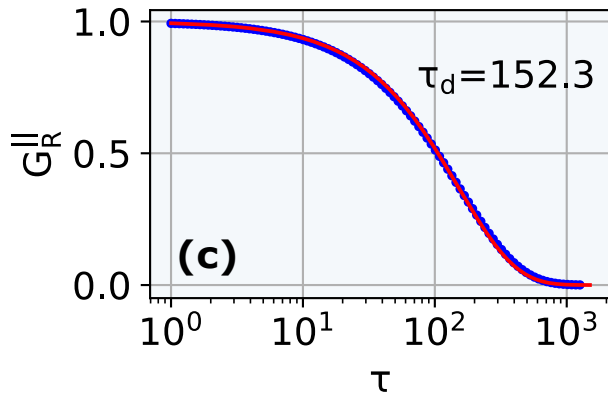

Supplement: Supplementary file 1 — nn3c03392_si_001.zip [file nn3c03392_si_001.zip › S_Pecora_functions.pdf]

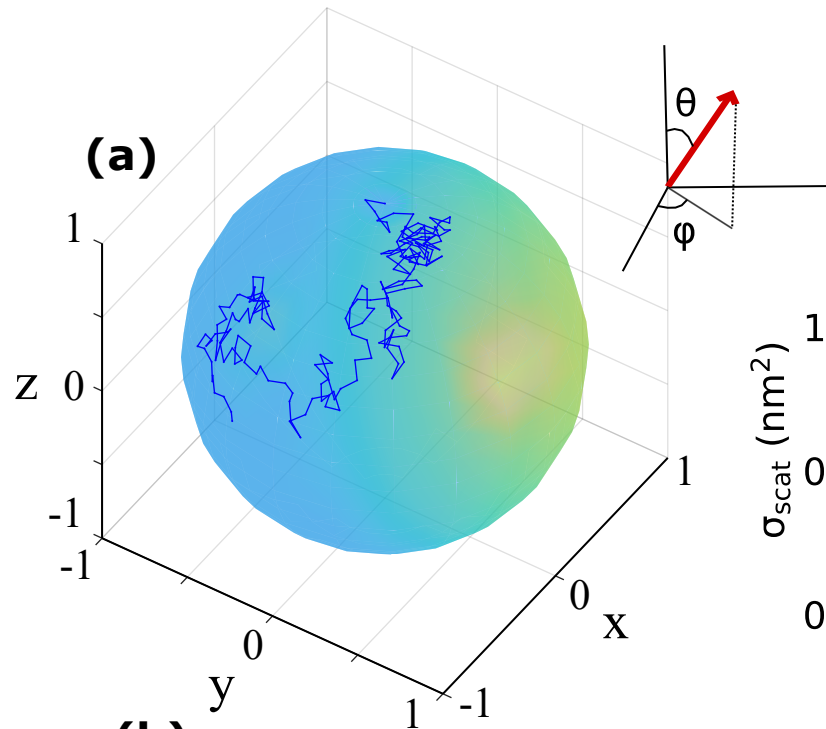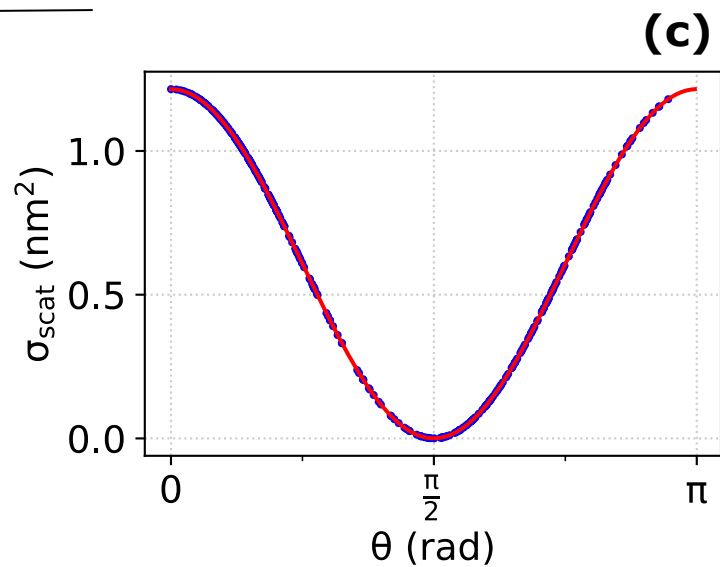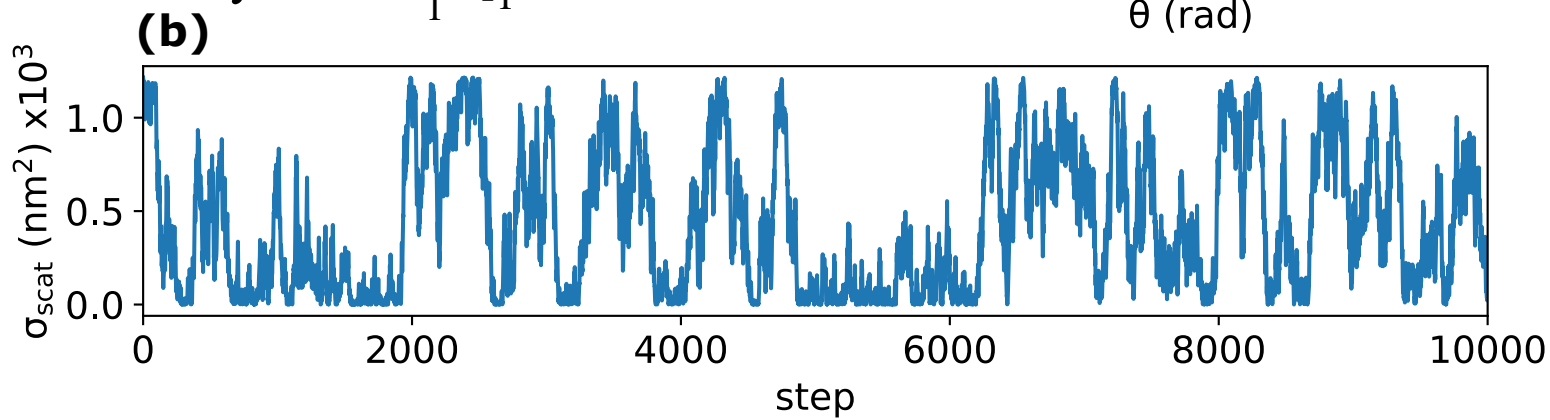

Supplement: Supplementary file 1 — nn3c03392_si_001.zip [file nn3c03392_si_001.zip › S_Random walk.pdf]

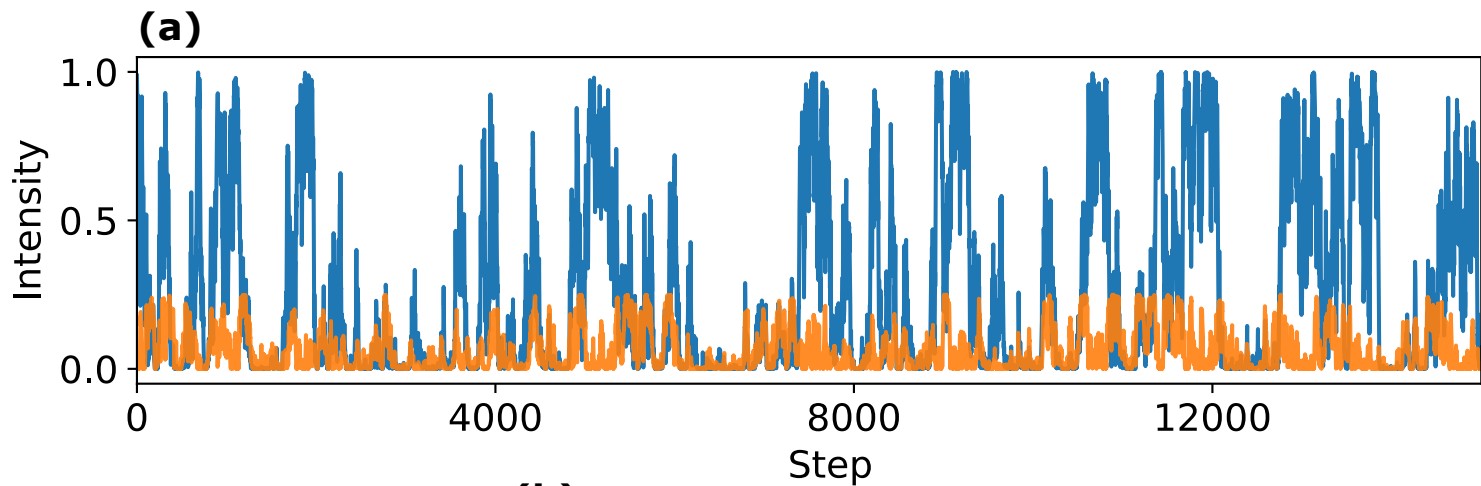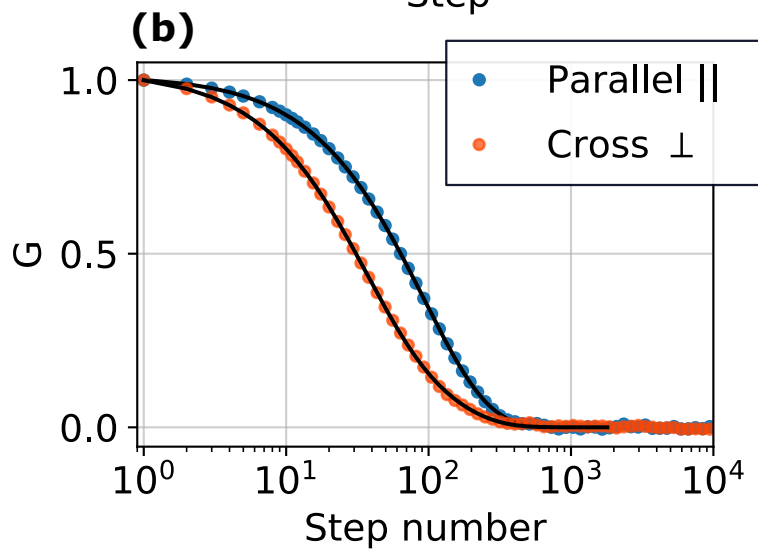

Supplement: Supplementary file 1 — nn3c03392_si_001.zip [file nn3c03392_si_001.zip › S_cross_parallel_walk.pdf]

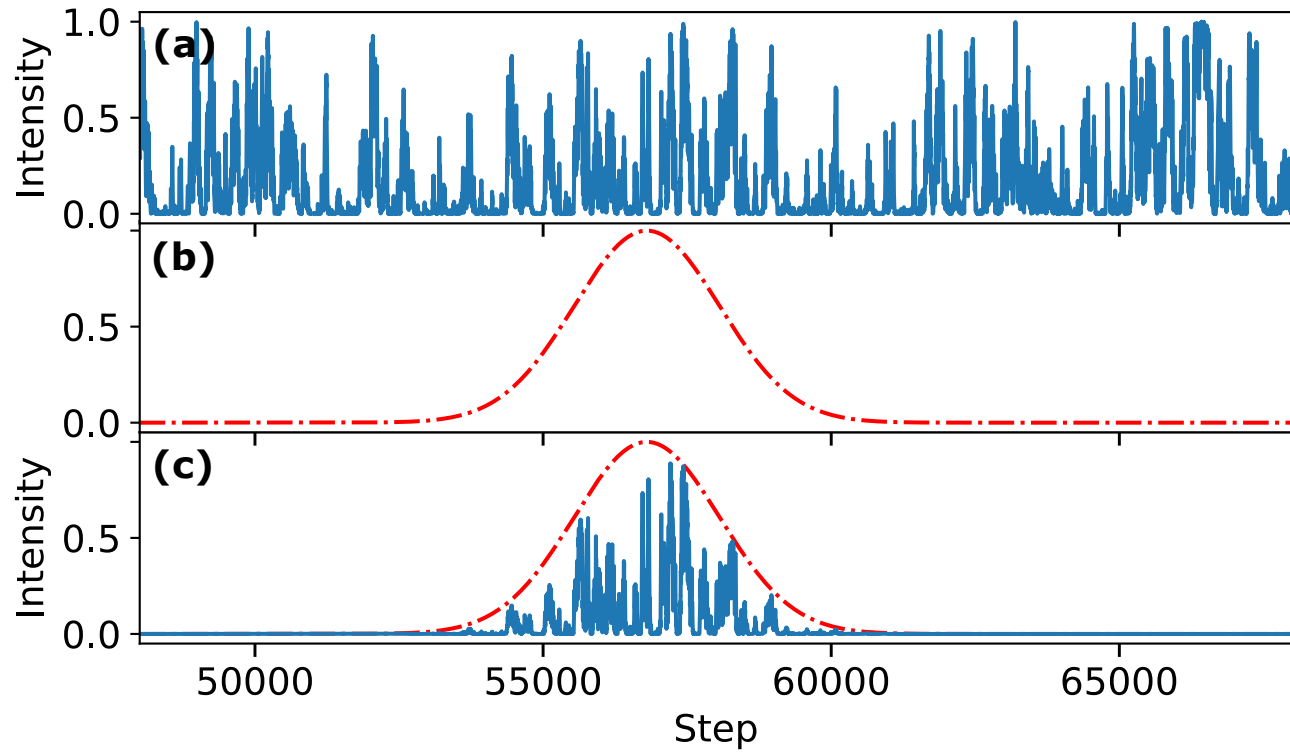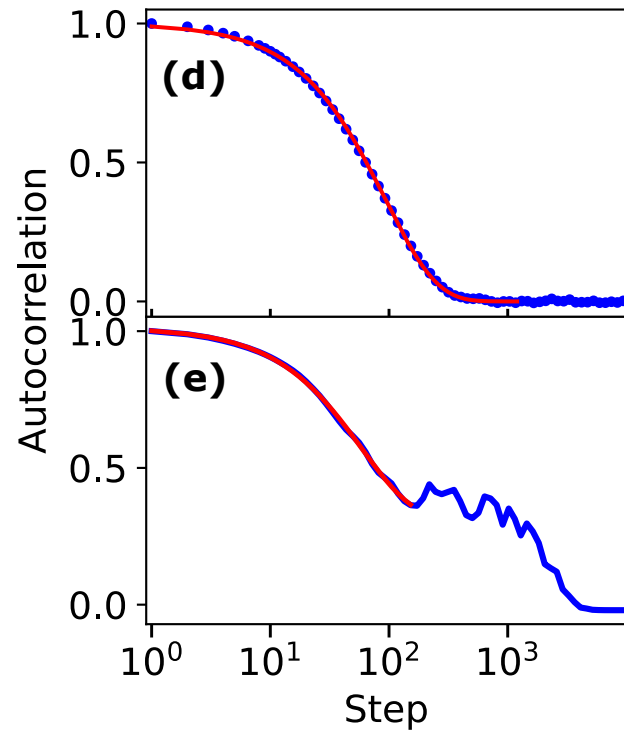

Supplement: Supplementary file 1 — nn3c03392_si_001.zip [file nn3c03392_si_001.zip › S_random_walk_gaussian.pdf]

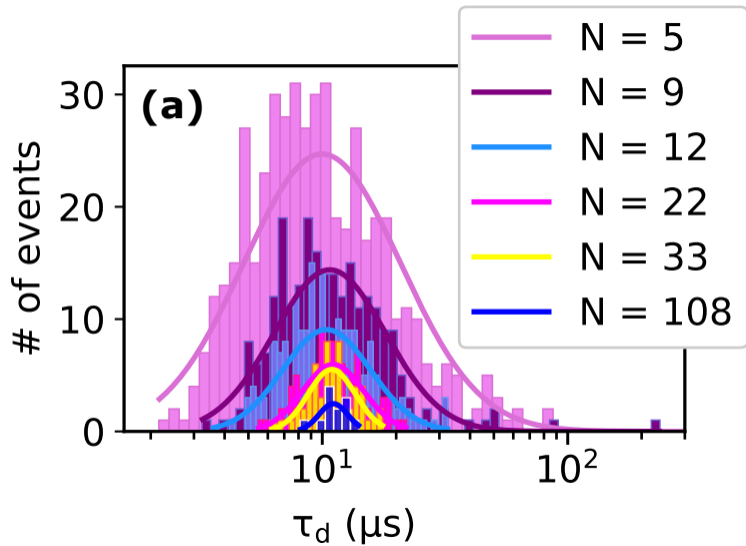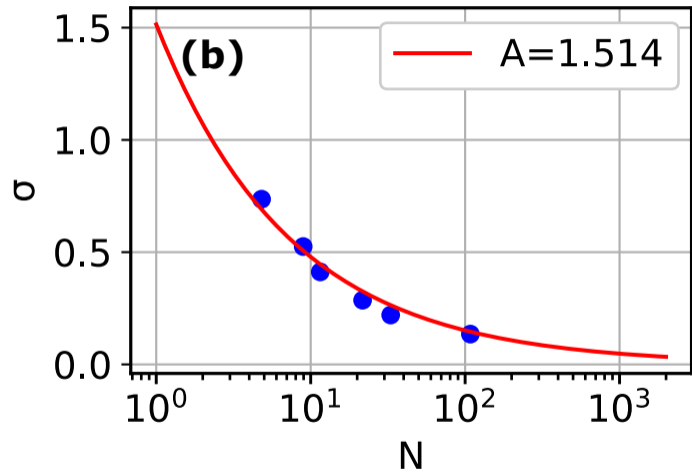

Supplement: Supplementary file 1 — nn3c03392_si_001.zip [file nn3c03392_si_001.zip › S_Random_walk_hist.pdf]

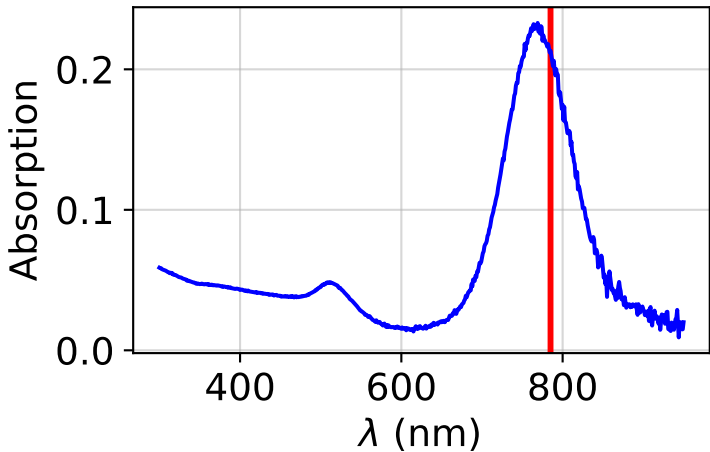

Supplement: Supplementary file 1 — nn3c03392_si_001.zip [file nn3c03392_si_001.zip › S_GNR_spectrum.pdf]

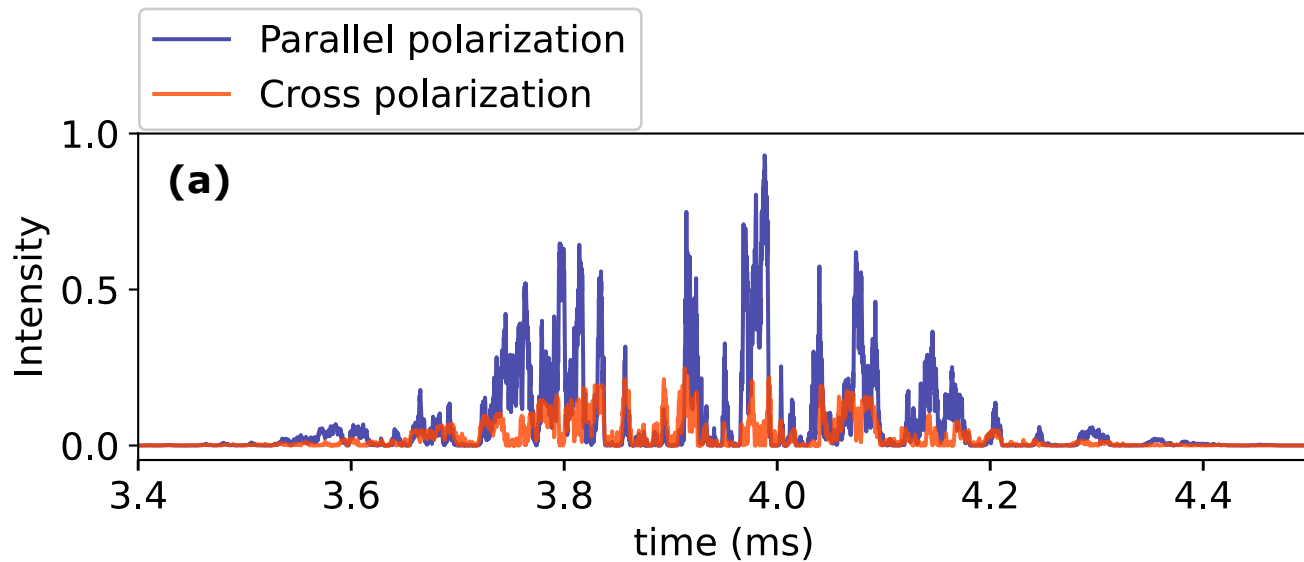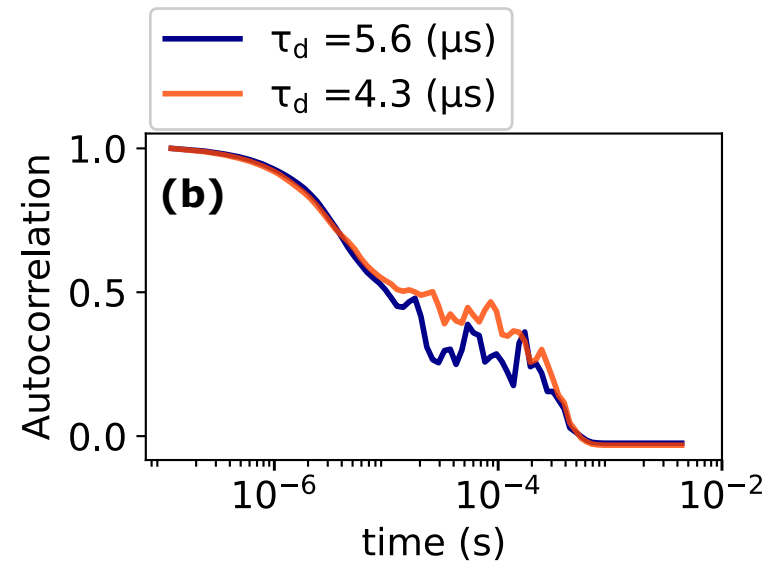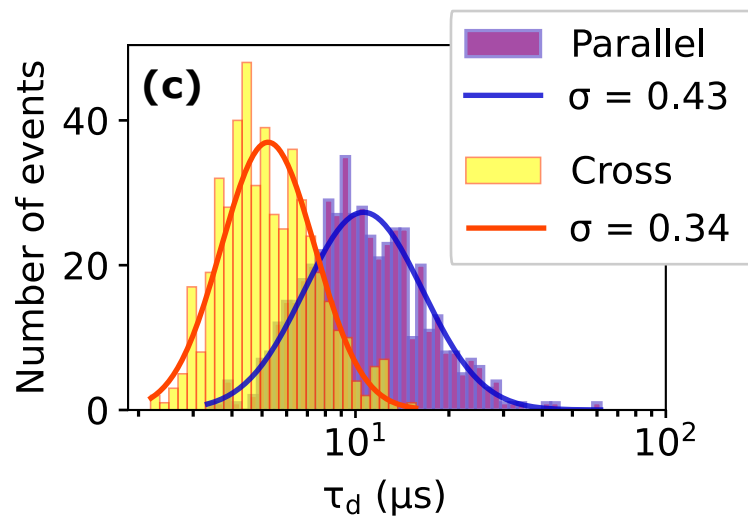

Supplement: Supplementary file 1 — nn3c03392_si_001.zip [file nn3c03392_si_001.zip › S_cross_parallel gaussian random walk.pdf]

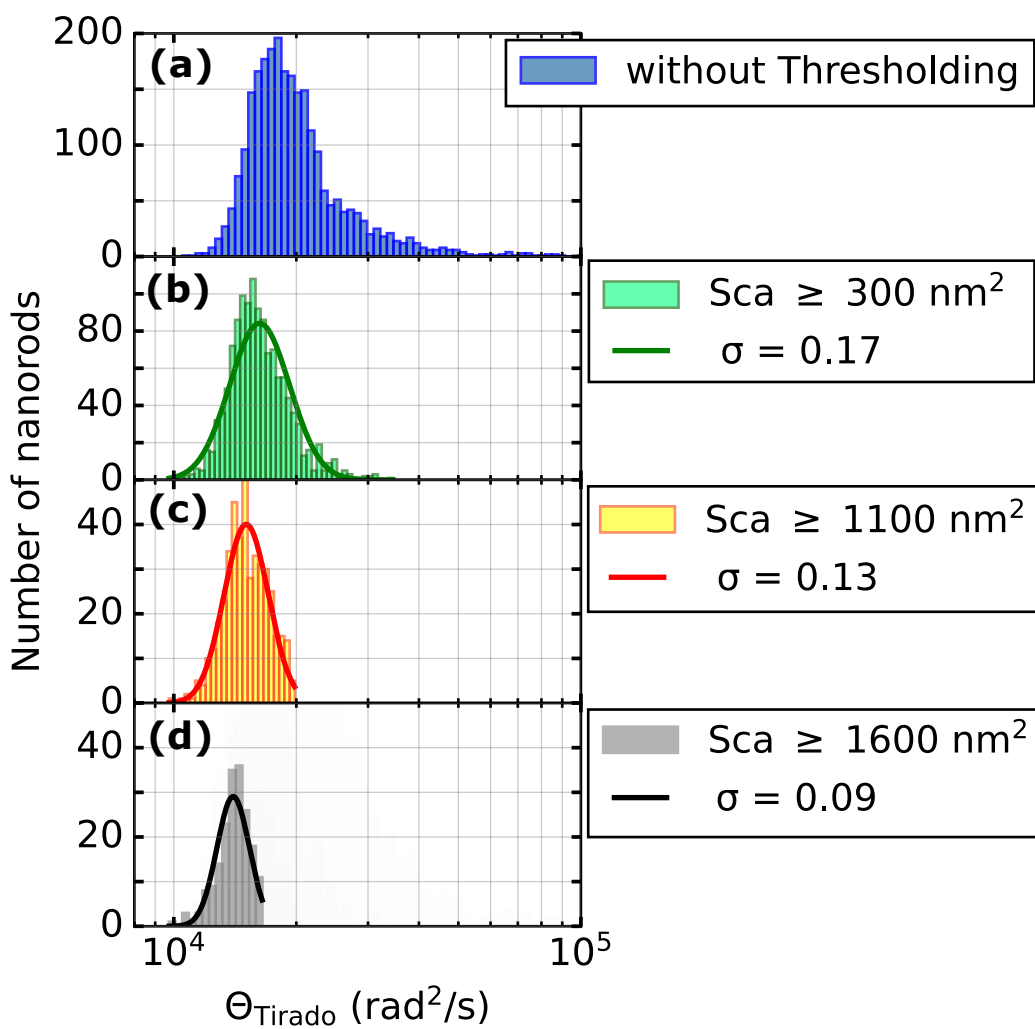

Supplement: Supplementary file 1 — nn3c03392_si_001.zip [file nn3c03392_si_001.zip › S_TEM_higher_threshold.pdf]

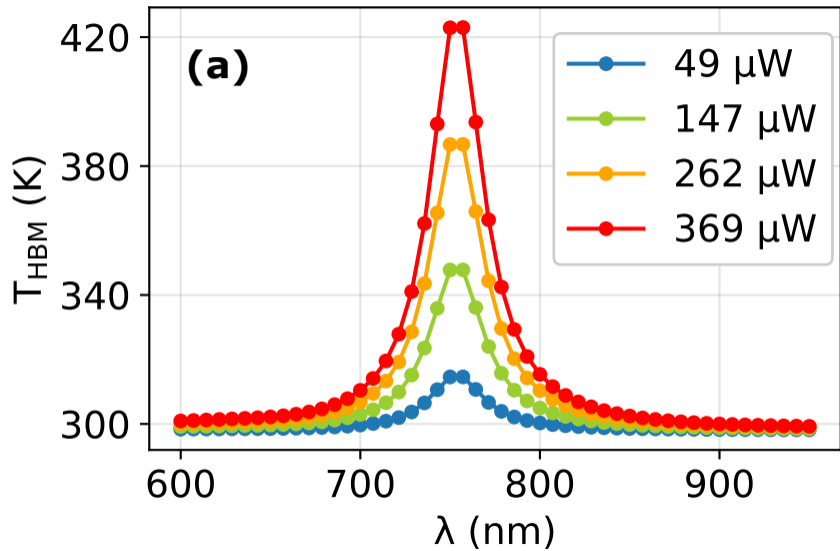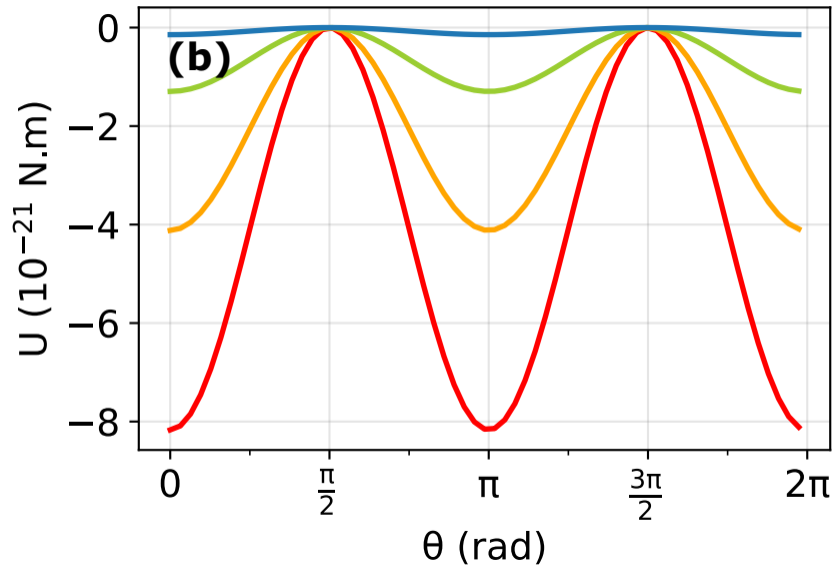

Supplement: Supplementary file 1 — nn3c03392_si_001.zip [file nn3c03392_si_001.zip › S_alpha.pdf]

Number of nanorods

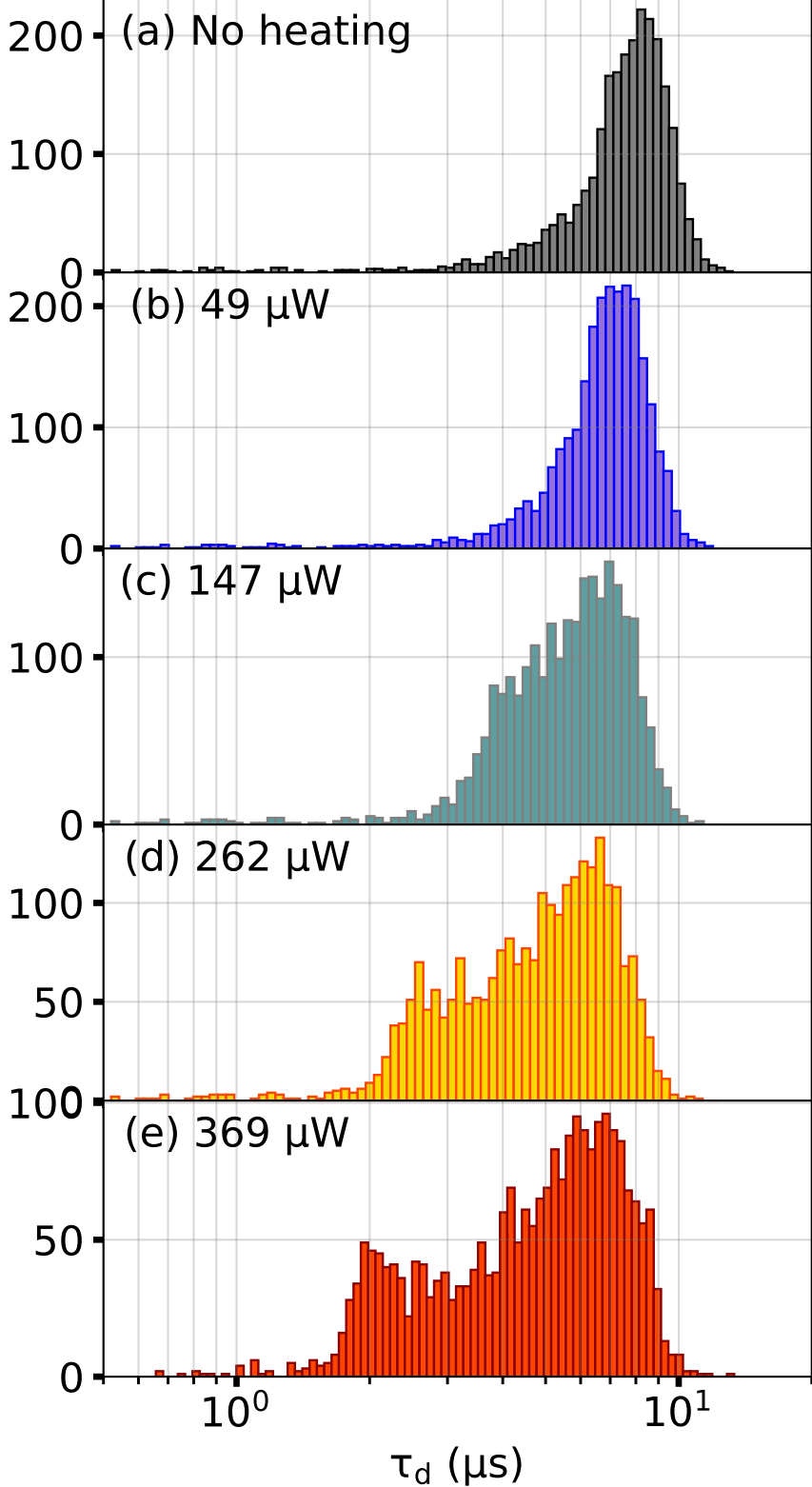

Supplement: Supplementary file 1 — nn3c03392_si_001.zip [file nn3c03392_si_001.zip › S_HBM_simulation.pdf]

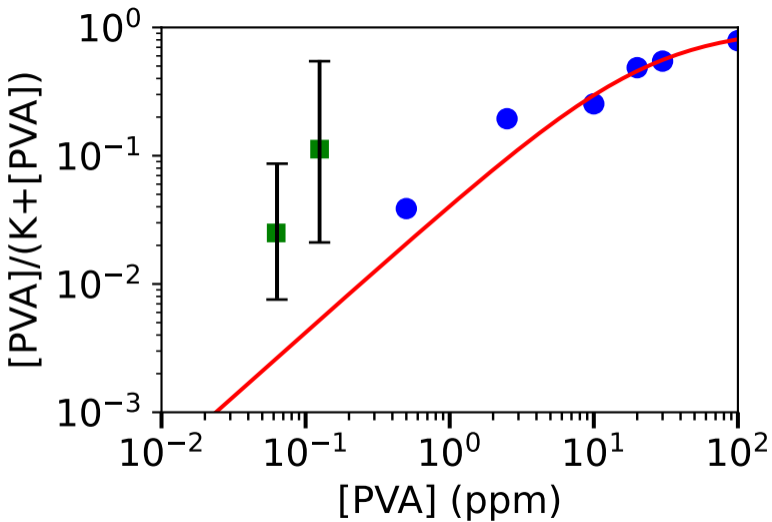

Supplement: Supplementary file 1 — nn3c03392_si_001.zip [file nn3c03392_si_001.zip › S_PVA_Affinity.pdf]

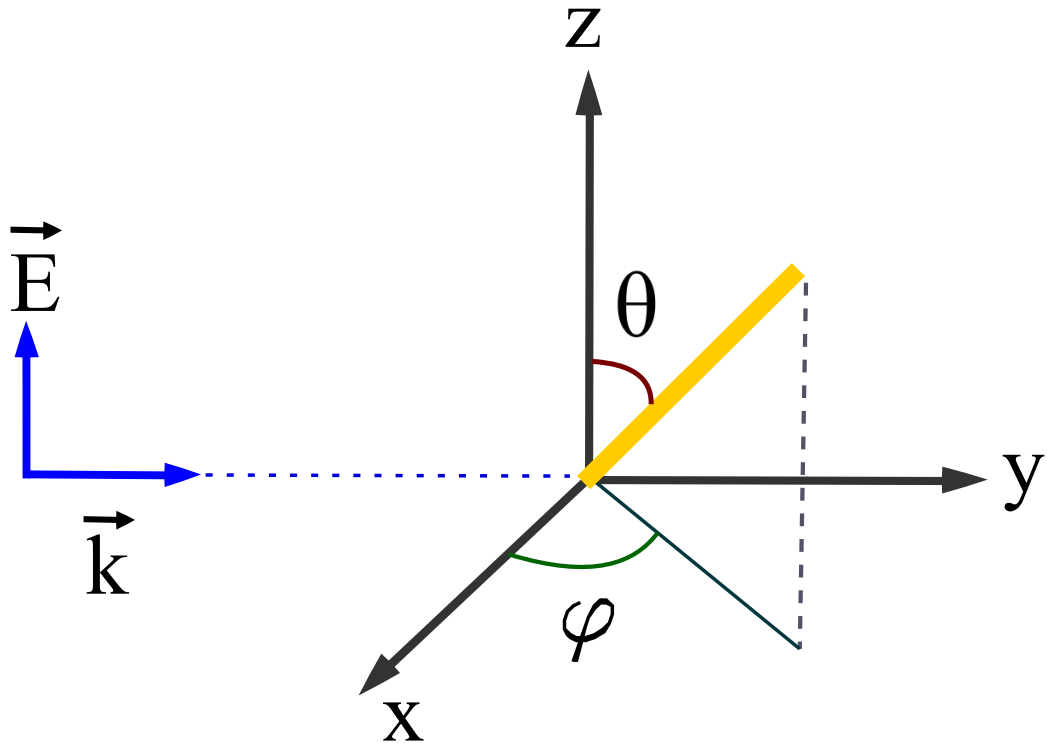

Supplement: Supplementary file 1 — nn3c03392_si_001.zip [file nn3c03392_si_001.zip › S_coordinate.pdf]

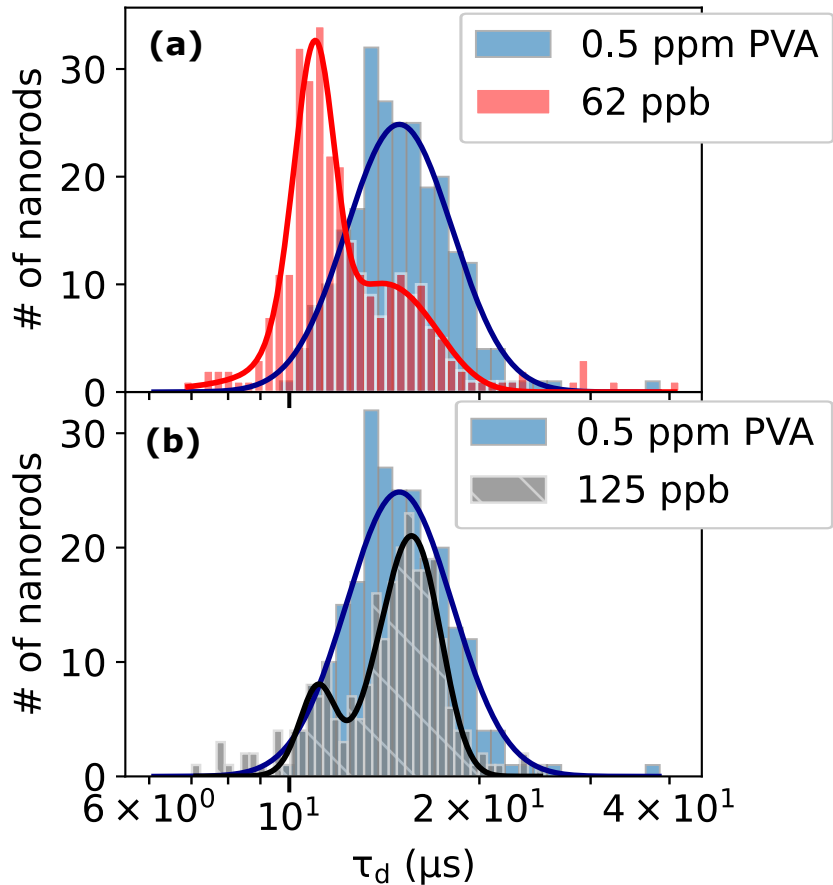

Supplement: Supplementary file 1 — nn3c03392_si_001.zip [file nn3c03392_si_001.zip › S_PVA.pdf]

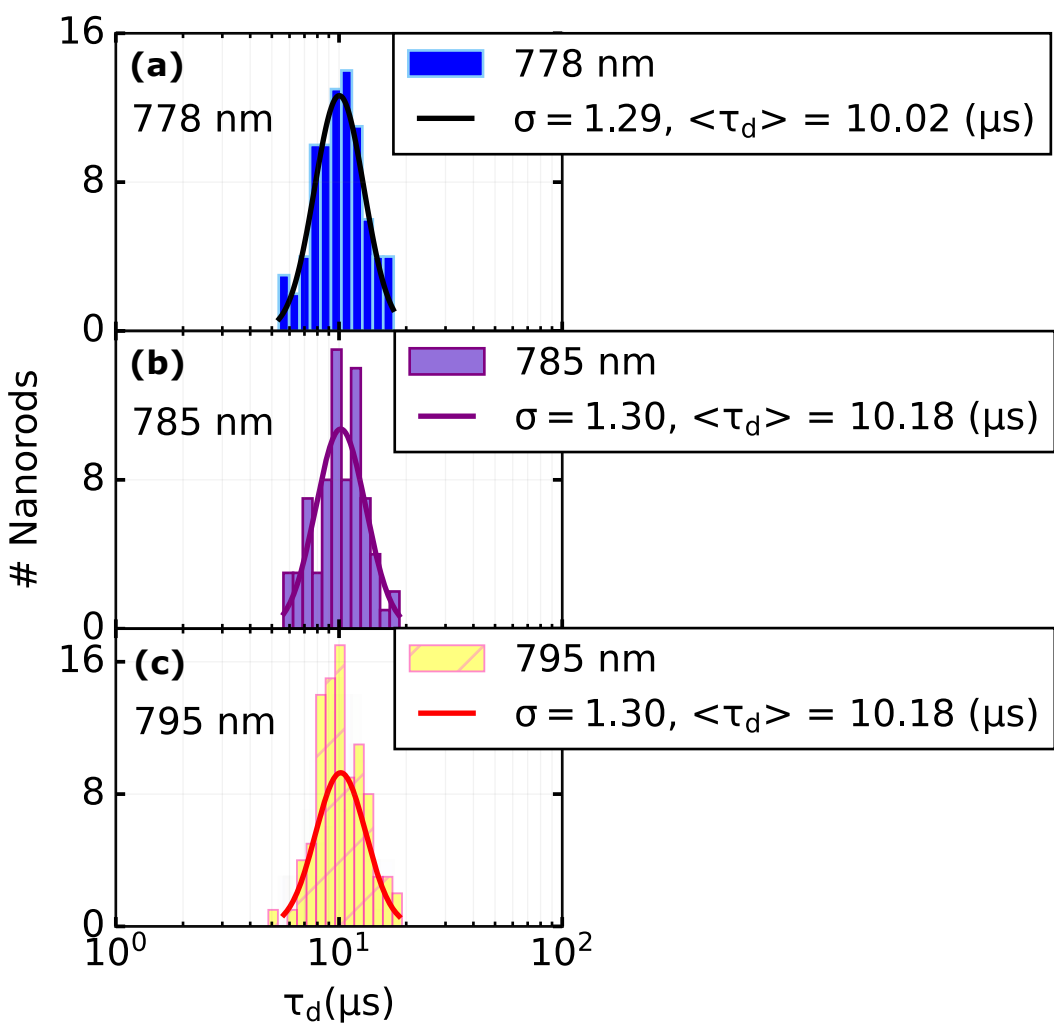

Supplement: Supplementary file 1 — nn3c03392_si_001.zip [file nn3c03392_si_001.zip › S_wavelengths.pdf]

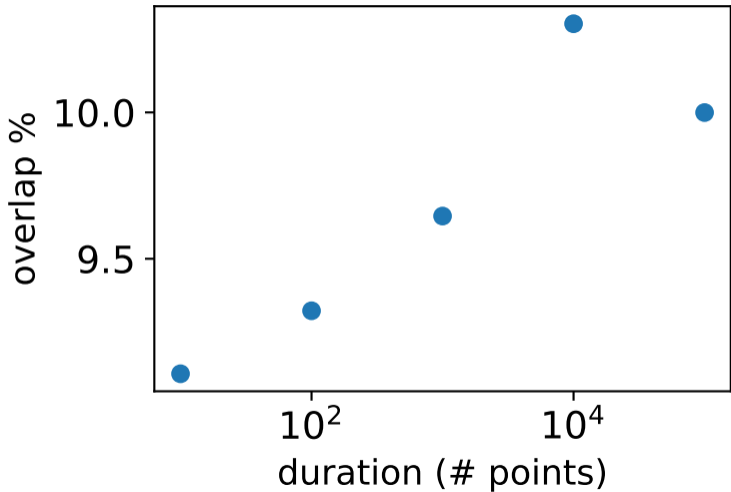

Supplement: Supplementary file 1 — nn3c03392_si_001.zip [file nn3c03392_si_001.zip › S_overlap.pdf]

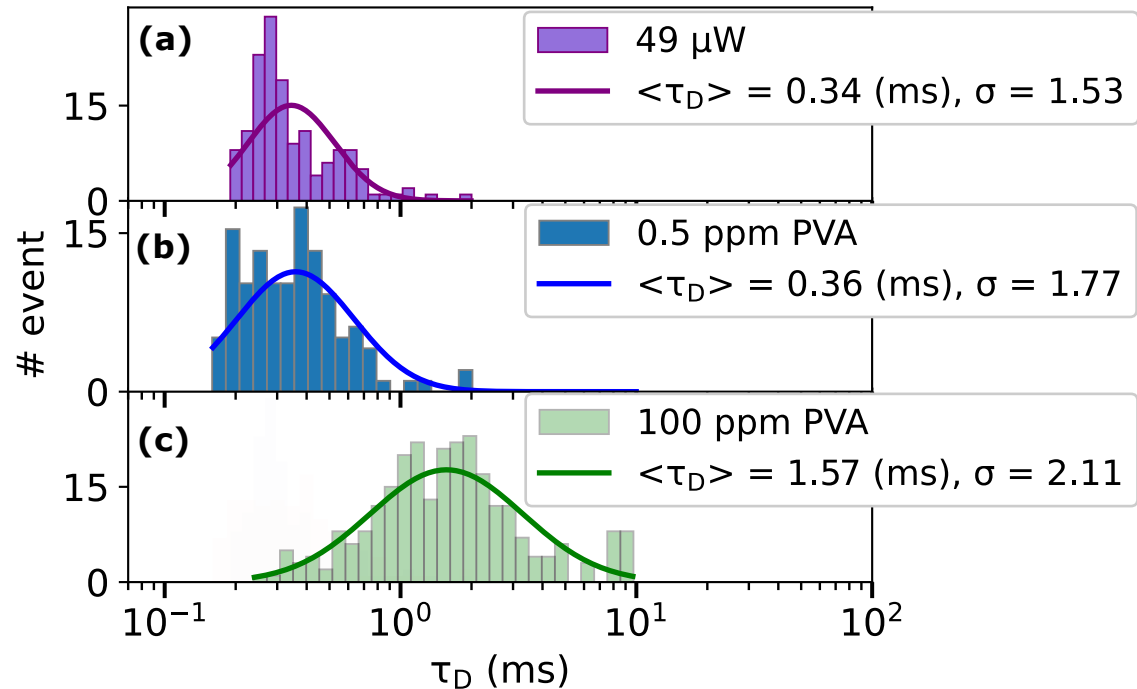

Supplement: Supplementary file 1 — nn3c03392_si_001.zip [file nn3c03392_si_001.zip › S_PVA_translation.pdf]

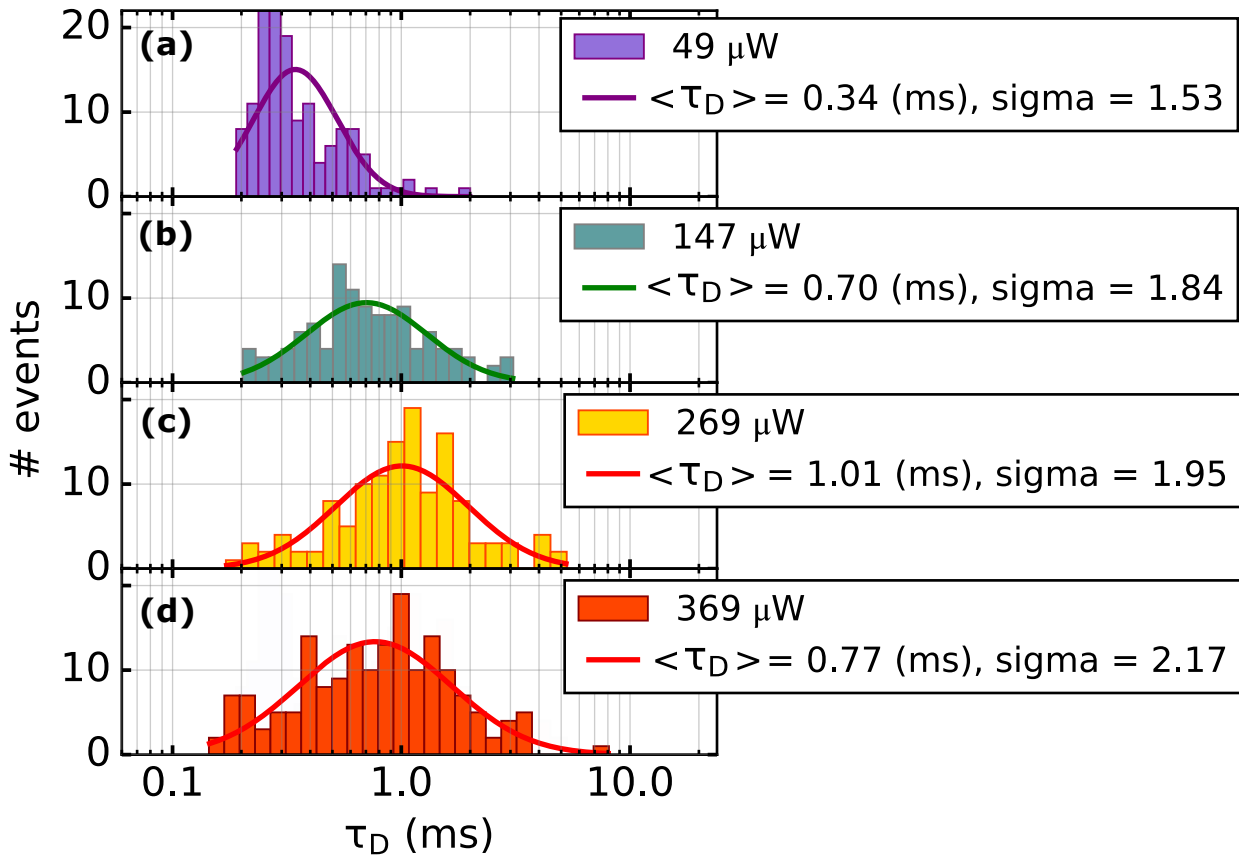

Supplement: Supplementary file 1 — nn3c03392_si_001.zip [file nn3c03392_si_001.zip › S_HBM_translation.pdf]

Number of nanorods

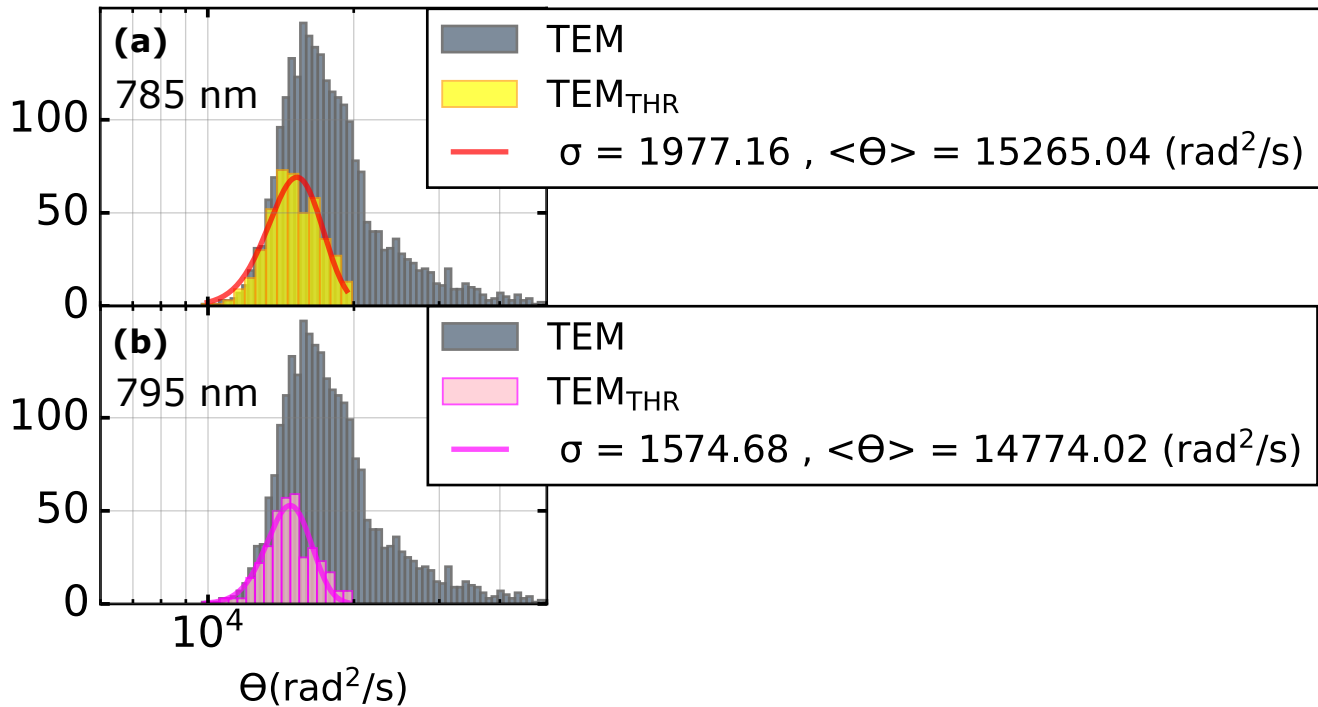

Supplement: Supplementary file 1 — nn3c03392_si_001.zip [file nn3c03392_si_001.zip › S_LSPR_785_795.pdf]
