# Supplementary material for: Burst-by-Burst Measurement of Rotational Diffusion at Nanosecond Resolution Reveals Hot-Brownian Motion and Single-Chain Binding
Source: ACS Nano. 2023 Jun 23;17(13):12684–92. doi: 10.1021/acsnano.3c03392 (PMC10339794; doi:10.1021/acsnano.3c03392)
Supplement: Supplementary file 2 — nn3c03392_si_002.pdf [file nn3c03392_si_002.pdf]

Supporting Information: Burst-by-Burst Measurement of Rotational Diffusion at Nanosecond Resolution Reveals Hot-Brownian Motion and Single-Chain Binding

Nasrin Asgari,[†] Martin Dieter Baaske,[†,‡] and Michel Orrit[*,†]

[†]*Huygens-Kamerlingh Onnes Laboratory, Leiden University, Postbus 9504, 2300 RA Leiden, The Netherlands*

[‡]*Max Planck Institute of Biophysics, Max-von-Laue-Str. 3, 60438 Frankfurt am Main, Germany*

E-mail: orrit@physics.leidenuniv.nl

S1 TEM

To determine the physical dimensions of GNR sample we have performed TEM microscopy. Transmission electron microscopy (TEM) images were taken at a magnification of $73,000$. GNR samples we prepared on a EM grid (CF200-Cu from Electron Microscopy Sciences). TEM example images are shown in fig. S1 and the distributions of the measured dimensions are depicted in fig. S2.

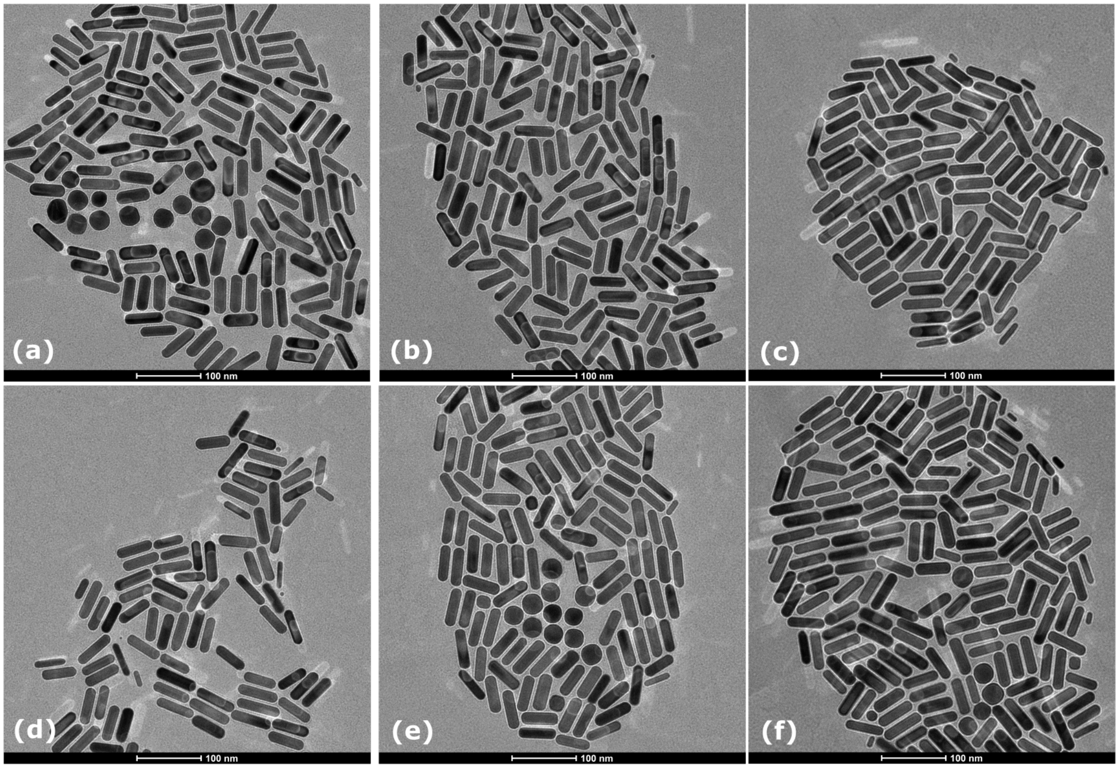

Figure S1: Transmission electron microscopy (TEM) images of the GNRs that have been used in our measurement. Scale bar: 100 nm.

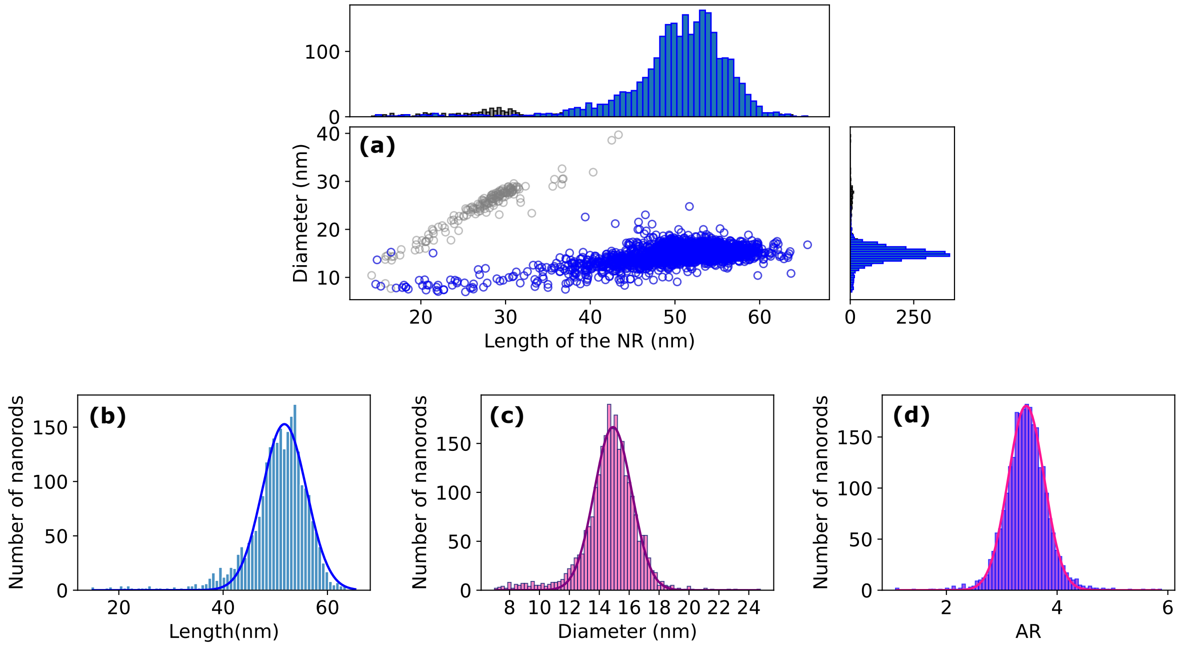

Figure S2: **a)** Scatter plot of GNR dimensions (2,680 particles) determined from TEM images showing two groups: nanorods (blue circles) and spheres (gray circles). Spheres are excluded from further analysis. **b)** Histogram of GNR lengths, solid line: Gaussian fit with mean value 51.7 nm and standard deviation of 4.2 nm. **c)** Histogram of diameter distribution of GNRs with fitted Gaussian curve. Mean diameter 14.9 nm and standard deviation 1.2 nm. **d)** Histogram of aspect ratios (AR) with Gaussian fit (solid line): Mean value 3.4 and standard deviation 0.3.

S2 Estimation of events overlap

To estimate the probability for one event overlapping with another we performed Monte-Carlo Simulations. Specifically we simulate traces with 10^6 points length and randomly seed events with a given duration into these traces until 10 percent of all points (as found experimentally) are occupied by at least one event. We then determine the ratio of the numbers of events that possess occupancy ≥ 2 and the total number of events. We run each simulation 30 times to minimize statistical noise. We find that in this situation 10% of detected events will result from the overlap of at least two events.

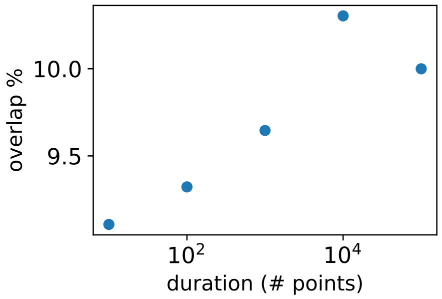

Figure S3: Fractions of events that overlap with at least one more event for five different event durations.

S3 Simulations of rotational diffusion

In latter sections of this SI we want to determine how several experimental conditions would influence the analysis, in particular the determination of rotational diffusion times. Such experimental parameters are different polarizer configurations but also the length of events as determined by the analyte's translational diffusion properties and the measurement geometry (detection volume). In order to gain a basic understanding of the influence of these parameters we have performed simulations of rotational diffusion, which we describe in the following:

In order to understand how the scattering signal changes according to the random orientation of a GNR, we simulated the orientation of the rod's long axis with a random walk on the surface of a unit sphere. To perform the random walk, we generate a small rotation vector $\hat{u}$ with a random direction determined by two random numbers η_1 and η_2 uniformly distributed between 0 and 1, giving isotropically distributed directions with polar ($\theta = [-\pi/2,\ \pi/2]$) and azimuthal ($\phi = [0,\ 2\pi]$) angles according to:

$$\theta = cos^{-1}(2\eta_1 - 1)$$
$$\phi = 2\pi\eta_2 \tag{1}$$

and determine a unit vector

$$\hat{u} = \begin{pmatrix} sin\theta \ cos\phi \\ sin\theta \ sin\phi \\ cos\theta \end{pmatrix}. \quad (2)$$

Rotation of the initial vector r_i by a small angle (ω) around vector $\hat{u}$ gives a shift vector

$$\vec{\Delta r_i} = \hat{u} \times \vec{r}\omega, \quad (3)$$

and the next position $\vec{r_{i+1}} = \vec{r_i} + \vec{\Delta r_i}$. We normalize this vector to avoid numerical errors. As starting value we use $\vec{r_0} = [0,\ 0,\ 1]$. We have found that $\omega = 0.1$ provides a good enough resolution with a reasonable number of steps to simulate long rotational diffusion events. To allow for direct correlation with our measurements one may convert step size to time units by using the conversion factor 0.12 µs/step, which was determined by comparing the autocorrelation decay time of random walk simulations with the average experimentally determined decay times.

S3.1 Rotational correlation function with polarization analysis of the detection

We want to investigate the influence of the incident and analyzed polarization configuration on the autocorrelation decay. For this we consider random orientations of the GNR axis (θ, ϕ) as described above and compute the respective intensity traces (neglecting the numerical aperture of the objective). We consider the GNR with polarizability along its main axis and write the components of polarizability $\overleftrightarrow{\alpha}$ relative to the rotated coordinates as $\overleftrightarrow{R}^T \overleftrightarrow{\alpha} \overleftrightarrow{R}$ where $\overleftrightarrow{R}$ is the rotation matrix in 3D. Then, by considering $\hat{y}$ as the optical axis and $E_0\hat{z}$ as the incident electric field (fig.S4), we find the scattered field as:

$$\vec{E_s} \propto (E_0\alpha) \begin{pmatrix} sin\theta cos\theta sin\phi \\ sin\theta cos\theta cos\phi \\ cos^2\theta \end{pmatrix}. \tag{4}$$

Consequently, the detected intensity in parallel ($I_{||}$) and cross ($I_\perp$) polarization configurations can be written as:

$$\begin{aligned} I_{||} &\propto cos^4\theta \\ I_\perp &\propto sin^2\theta\ cos^2\theta\ sin^2\phi. \end{aligned} \tag{5}$$

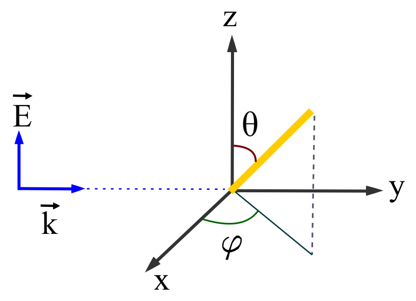

Figure S4: Polar θ and azimuthal ϕ angles are shown in cartesian coordinates of the optical measurement. $\hat{y}$ is the optical axis (xz is focal plane), the incident electric field is along $\hat{z}$ with propagation direction $\vec{k}$ along $\hat{y}$.

From these simulated intensity traces, we then compute the autocorrelation curves and fit them with a double exponential decay as:

$$G(\tau) = c_2 e^{-\Gamma_2 \tau} + c_4 e^{-\Gamma_4 \tau}, \tag{6}$$

Then we can compare the obtained fitting parameters with Pecora's decays (eq. 2 main manuscript). Examples of simulated traces and autocorrelations are shown in fig. S5a and b, respectively. For both configurations we find excellent agreement of our fitted values with Pecora's model (see tables. 1 and 2, below).

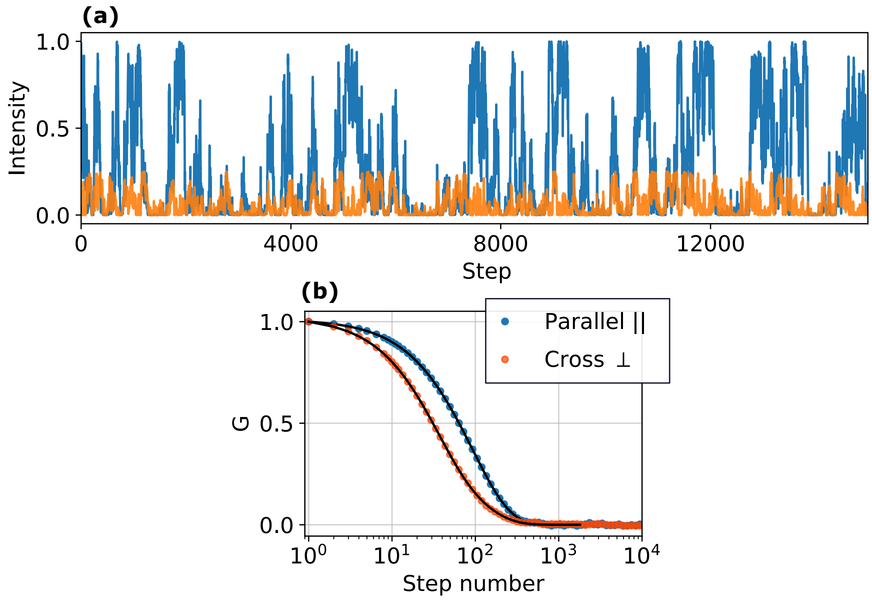

Figure S5: **a)** Excerpts of simulated intensity traces (blue:parallel, orange:crossed configuration) versus step number of the random walk. **b)** The autocorrelation functions (dots) of the intensity traces in (**a**), calculated for 2×10^6 steps. Solid lines: Fits to double-exponential decays.

Table 1: Comparing the values obtained from fitting a double-exponential decay to simulation data with the coefficients expected from Pecora's theory for parallel-polarized configuration.

Parallel polarization			
	Γ_4/Γ_2	Normalized c_2	Normalized c_4
Theory	3.33	0.91	0.08
Simulation	3.83	0.93	0.06

Table 2: Comparing the values obtained from fitting a double-exponential decay to simulation data with the coefficients expected from Pecora's theory for cross polarization.

Cross polarization			
	Γ_4/Γ_2	Normalized c_2	Normalized c_4
Theory	3.33	0.35	0.64
Simulation	3.33	0.34	0.65

As our experimental data sets are too noisy to allow for fitting of double-exponential decays, we fit them with single exponentials. In order to find the relation between the decay times in Pecora's double-exponential decays with the decay times obtained from single-exponential fits in either configurations, we fit single exponential decays directly to Pecora's functions (eq. 2 main manuscript) as shown fig. S6. We find a ratio of $\tau_{d,||}/\tau_{d,\perp}$ between decay times obtained in parallel and cross configuration, in good agreement with the experimental values of 2.06 (and also with the simulation value of 1.96, by fitting single exponential functions to the simulated autocorrelations in fig. S5b).

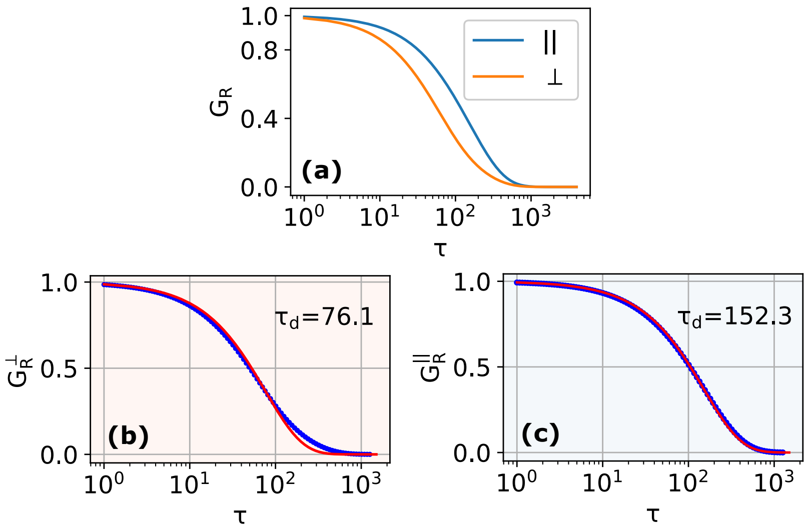

Figure S6: **a)** Rotational correlation functions from Pecora's model for a dipole for parallel (blue) and crossed (orange) polarization configurations. **b)** Rotational correlation function of the cross polarization configuration with a single-exponential fit (red) $y = e^{-1/\tau_d x}$ gives a decay time of τ_d=76.1 steps. **c)** Rotational correlation function of the parallel polarization and single-exponential fit with a decay time of τ_d=152.3 steps. By comparing these decay times, we see that the crossed polarization decays 2.00 times faster than the parallel one, which agrees well with the experimental histograms in the main text (fig. 2e, f) (also, fitting a single exponential to the simulated autocorrelations in fig. S5b yields the ratio of 1.96 for $\tau_{d||}/\tau_{d\perp}$).

S3.2 Effect of event length on accuracy of the decay times

We investigate the clipping of the rotational diffusion trace by the duration of each event, which is limited by translational diffusion of the rod through the confocal volume. Short

enough events will lead to a broadening of the rotational diffusion histogram due to finite sampling of the trace. As a simple model of clipping by translational diffusion, we multiply the rotational diffusion trace by a Gaussian envelope, which simulates the dwell time of the rod in the confocal volume. The Gaussian envelope here is just meant as a convenient way of varying the sampling time of rotational diffusion. In real measurements, however, the random diffusion path would lead to more statistical fluctuations, which we ignore here. Fig. S7 is an example of how we simulate the events. To see the effect of statistical fluctuations clearly, here, we have considered the events much shorter than the translational diffusion through the confocal volume.

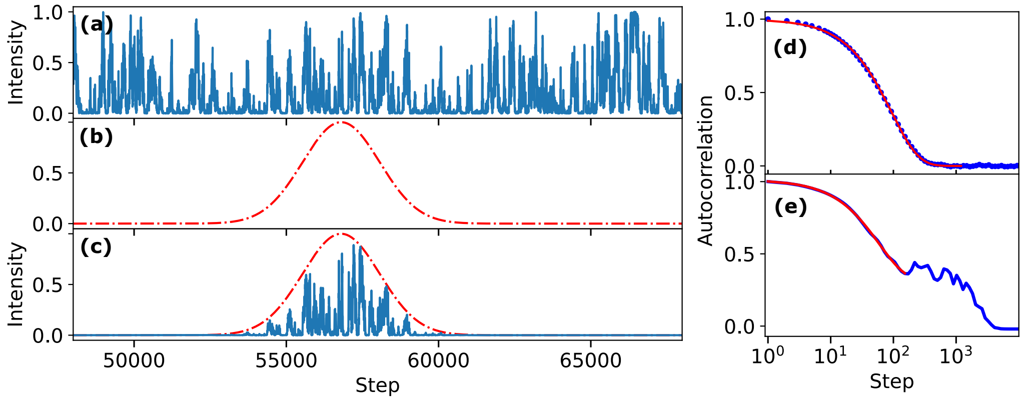

Figure S7: **a)** Simulated intensity time trace for parallel-polarization configuration, according to Equation 5. **b)** A Gaussian curve with 800 steps as standard deviation. **c)** Multiplication of the trace in (a) with the Gaussian curve in (b), which clips the rotational diffusion trace to the duration of the translational event. **d)** Autocorrelation of the unclipped intensity trace in (a) with a single exponential curve fit (red) with decay time of 93 steps. **e)** Autocorrelation of the simulated clipped event in (c), showing two distinct decay times, similar to our experimental data. A single-exponential fit (red) to the fast decay gives a decay time of 65 steps, significantly shorter than the unclipped trace's time.

By increasing the width of the events, we expect to have higher statistics of sub-bursts in each event which yields a narrower histogram of rotational diffusion times (or τ_d). Here, we investigate the influence of the event duration on the width of the histogram. We introduce as a parameter the ratio N of the event duration to the tumbling time as equation 7, which is roughly the number of sub-bursts in an event:

$$N = \frac{\tau_T}{\tau_d}, \tag{7}$$

where τ_T is the translational diffusion time through the confocal volume (corresponding to the duration of events) and τ_d is the decay time of the rotational autocorrelation. Fig. S8a shows the simulated histograms for different numbers of sub-bursts (N). We have fitted a log-normal function:

$$Log - normal(\mu, \sigma^2) = \frac{1}{x\sigma\sqrt{2\pi}} \exp\left(-\frac{(lnx - \mu)^2}{2\sigma^2}\right), \tag{8}$$

to each histogram, where μ and σ respectively are expected value (or mean) and standard deviation of the variable's natural logarithm, and σ has been plotted as a function of N in fig. S8b. Increasing N reduces the width of the histogram, according to the central limit theorem:

$$\sigma = \frac{A}{\sqrt{N}}, \tag{9}$$

where A is a fit parameter and σ is the standard deviation of the log-normal distribution in equation 8. In our confocal measurement, a typical event duration is around 2 ms. By considering our experimental value of 11 µs as the mean rotational diffusion time, we find $N \geq 180$, even higher than the highest $N = 108$ in fig. S8b. Thus, the large number of sub-bursts per event in our experiments provides for a very accurate estimate of the rotational diffusion for each individual event.

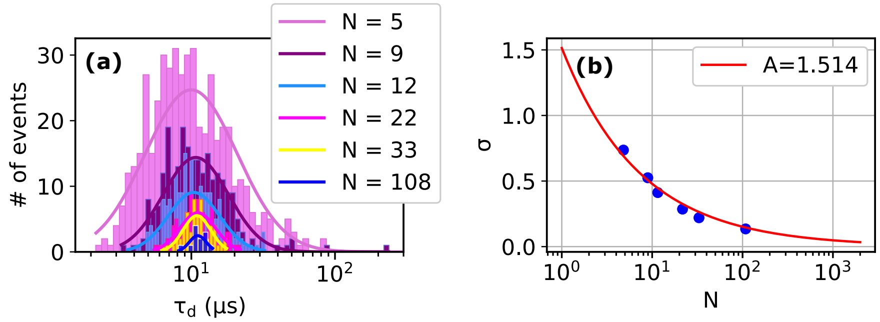

Figure S8: **a)** Histogram of simulated decay times (fig. S7e) for different numbers of sub-bursts N. The histograms are fitted with a log-normal distribution function. By having more sub-bursts within each event, the rotational diffusion time can be determined more precisely. **b)** The standard deviation (σ) of the log-normal fitted curve of each histogram versus N (blue dots). The plot is fitted by equation 9 where A is the fit parameter.

S3.3 Influence of polarization configuration on decay time accuracy

In our experiments, we found that τ_d and Θ-distributions obtained in cross-polarized configuration are narrower than their parallel-polarized counterparts. Therefore, here we compare simulated sub-bursts in parallel- and cross-polarization as obtained from the same random walks and repeat the analysis as performed above for different Gaussian windows (fig. S9). We find that the cross-polarized configuration provides narrower distributions due to its overall faster fluctuations and therefore higher number of sub-bursts. This difference, however, quickly becomes very small for longer event durations and is therefore negligible in our measurements. The main narrowing effect in the cross-polarized configuration is due to stronger selection by the LSPR, as discussed below.

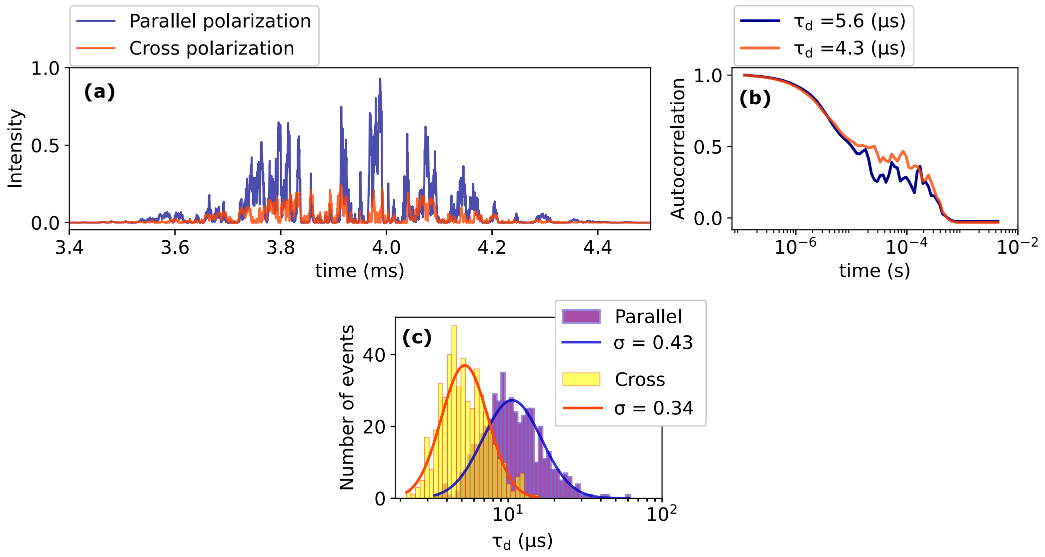

Figure S9: **a)** Simulated events for parallel (purple) and crossed (orange) polarization configurations (with $N = 9$). **b)** Corresponding autocorrelations of these events which show faster decay for crossed polarization in comparison to the parallel one. **c)** Histograms of decay times for the specific event duration. Solid lines: Fits to log-normal functions with mean values of 10.6 µs and 5.2 µs for parallel and cross polarized excitation, respectively. The ratio of the mean values is 2.1, which is in agreement with the experimental value of 2.07.

S4 LSPR-based detection

In our measurements, detection of the GNRs is greatly facilitated by resonance of their Localized Surface Plasmon (LSP) with our probe laser at 785 nm. We select those GNRs which have a resonance wavelength close to that value. Here, we investigate the effect of selection by LSPR on the width of rotational diffusion histograms. We calculate the scattering cross sections σ_{scat} at 785 nm for many individual GNRs drawn at random from the population determined from TEM images (blue dots in fig. S2a), and supposing diameter and length of the rods to be independent random variables. The results are displayed in fig.S10, where each dot is color-coded by its scattering cross section in a length-diameter scatter plot. Because of a comparatively narrow distribution of diameters, we see that the distribution of GNRs in resonance with the laser is considerably narrower than the general distribution. The scattering cross sections of the best rods are more than 3 times larger than

the average cross section. Only these best rods will be detectable optically, leading to a very narrow histogram. Such narrow histograms are the cornerstone of our method for detecting small changes in rotational diffusion constant. Such changes would be much more difficult to detect on the un-selected histogram (blue histogram in fig.S10c).

By comparing the simulated data with the experimental histogram of parallel polarization in fig.2h of the main text, we choose a minimum scattering cross section of 1100 nm^2 as detection threshold for the measured GNR bursts (fig. S10).

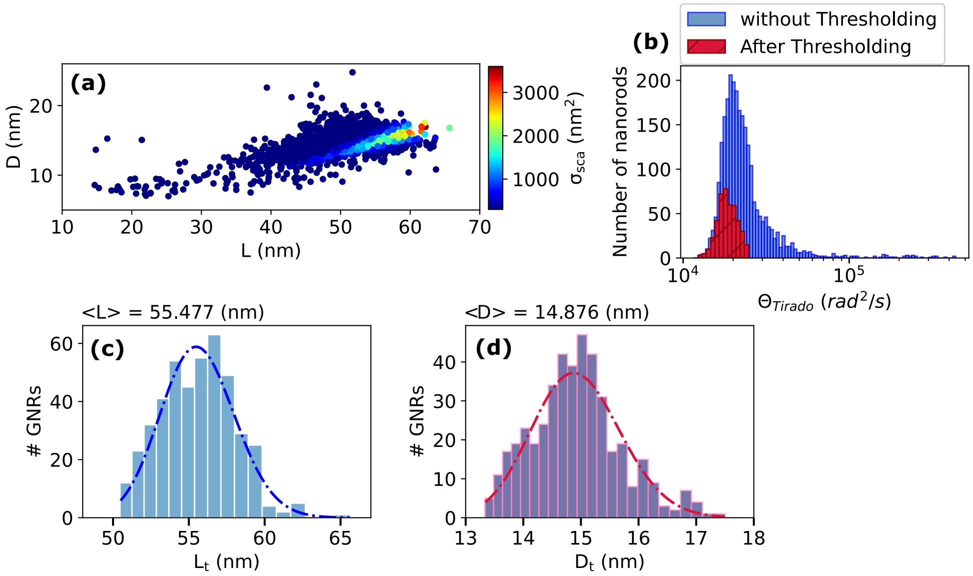

Figure S10: **a)** Distribution of GNRs from TEM images with color-coded scattering cross section. (same as blue population in fig. S2). The scattering cross sections have been calculated by MNPBEM,[1] for linearly polarized light along the main axis of each GNR and wavelength of 785 nm (same wavelength as our laser) in water as medium. To remove the nanorods which are out of resonance, we set a threshold on the scattering cross section σ_{sca} at (1100 nm^2). 440 GNRs have scattering cross section higher than the threshold. **b)** Histogram of calculated rotational diffusion coefficients of GNRs. In the blue histogram, all the GNRs have been considered and in the dark red histogram, only those with cross section higher than threshold have been considered. The red histogram is narrower than the blue one which indicates the selectivity of our method (based on localized surface plasmon resonance (LSPR)). The small difference of the mean values in these histograms is due to the small difference of the average LSPR peak in comparison to our laser wavelength (785 nm), as shown in fig. 1h in the main text. The blue and red histograms are equivalent to the gray and yellow histograms of fig. 2j in the main text. In that figure, a 2.2 nm CTAB (Cetyltrimethylammonium Bromide) layer has been considered. **c, d)** Histograms of lengths and diameters of GNRs after thresholding shown in (a) with mean values of $L_{mean} = 55.4$ nm and $D_{mean} = 14.8$ nm.

As mentioned in the main text, the main reason for the narrower histogram of the cross-polarized configuration in comparison to the parallel one (fig.2 g and h in the main text) is that the spectral selection of GNRs is even stricter, because of the much lower amplitude of scattering in the cross-polarized configuration. To check the effect of a stricter spectral selection, we compare the width of the calculated histograms for different cross section thresholds. After fitting a log-normal curve on the histograms and extracting σ as a function of the width of the histogram, we see the width decreases by increasing the threshold. Therefore, strengthening the LSPR selection further narrows down the histogram of rotational diffusion constants.

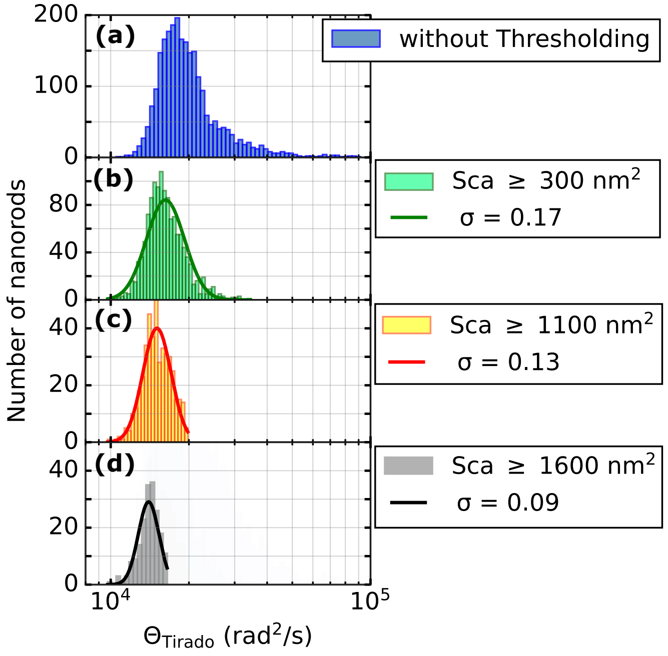

Figure S11: Calculated histograms of rotational diffusion coefficients Θ for different thresholds of scattering cross sections. **a)** Histogram of Θ from the whole population of GNRs from TEM images (whole population in fig. S10a) without any selection on the GNRs. **b)** Histogram of the selected population of GNRs with scattering cross section higher than 300 nm^2 and a log-normal fit. **c)** Histogram of Θ for selected GNRs with 1100 nm^2 as threshold, same population as fig. S10b. **d)** Histogram of Θ by setting 1600 nm^2 as the threshold. The width of the histograms decreases markedly as the threshold is raised. All the histograms have been plotted considering a 2.2 nm CTAB layer.

S5 The rotational diffusion times are independent of laser detuning from LSPR

Our measurement is biased to measure only a small fraction of the actual particles, those for which the laser is in resonance with their LSPR. Changes in experimental conditions might lead to selection of a different sub-population of rods, which couldn't be compared to the initial one. Here, we prove that a change of LSPR-laser detuning has no influence on the rotational diffusion times. To do this, we have measured rotational diffusion histograms at different laser wavelengths, thereby varying the detuning from the average LSPR. The results are displayed in fig. S12 for three different wavelengths, 778, 785 and 795 nm. All three histograms exhibit excellent overlap with only a minor shift of mean values on the order of 100 ns (2.5%), significantly less than the changes observed upon binding of a single PVA chain ($\approx 4\,\mu$s).

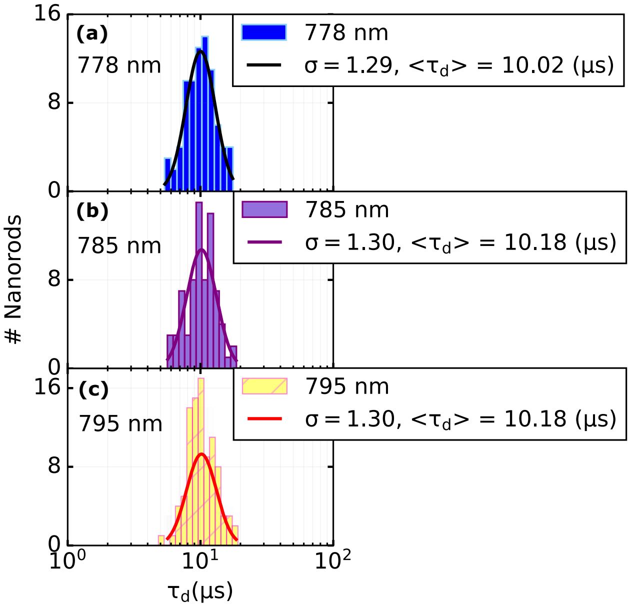

Figure S12: Histograms of decay times τ_d measured with different laser wavelengths. **a)** 778 nm, **b)** 785 nm and **c)** 795 nm. The histograms have been fitted with lognormal curves with mean value $\langle\tau_d\rangle$ and standard deviation σ.

Also, we did a calculation of the selected rotational diffusion coefficients for two differ-

ent incident wavelengths 785 nm and 795 nm and compare these thresholded populations in fig. S13. The histograms of rotational diffusion coefficients Θ overlap perfectly, which proves that biasing the plasmonic selection will not change the rotational diffusion coefficients.

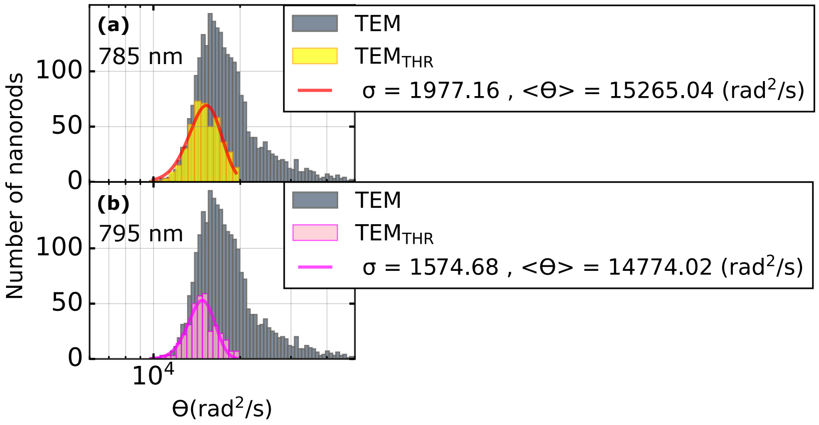

Figure S13: Calculated rotational diffusion coefficients for extracted dimensions of GNRs from TEM images (gray histograms). **a)** The yellow histogram is for 785 nm incident light and thresholding at a scattering cross section of 1100 nm^2; **b)** pink histogram for an incident wavelength of 795 nm, same thresholding.

S6 Rotational Hot Brownian motion (HBM)

S6.1 Theory and simulation

The temperature increase δT of a gold nanorod with length L and diameter D under illumination with light intensity (or irradiance) I is given by:[2]

$$\delta T = \frac{\sigma_{abs} I}{4\pi\kappa a_0 \beta}, \tag{10}$$

where δT is the temperature change of the nanoparticle $T_{NP} - T_0$, the correction factor β is $\beta \approx 1 + 0.096587\ ln^2(L/D)$ and σ_{abs} is the absorption cross section of the GNR. I is the power per unit area, κ is the thermal conductivity of the surrounding medium and a_0 is the radius of a sphere of equal volume.

A hot particle performing a translational Brownian motion carries a higher-temperature

halo with itself, characterized by an effective temperature T^x_{HBM}. A hot spherical particle with radius R has an effective translational diffusion coefficient D_{HBM} similar to the well-known Stokes-Einstein coefficient, but modified by a different temperature and the associated different viscosity, as:[3]

$$D_{HBM} = \frac{k_B T^x_{HBM}}{6\pi\eta^x_{HBM} R} \tag{11}$$

where k_B is Boltzmann's constant and η^x_{HBM} is an effective viscosity of the surrounding medium in this temperature gradient. The effective temperature and viscosity are determined so as to keep the above form of the Stokes-Einstein equation, by taking the temperature dependence of the viscosity into account.

For the case of rotational diffusion, the flow field is more localized around the particle which yields a higher effective temperature in comparison to the translational one:

$$T_0 \leq T^x_{HBM} \leq T_{HBM} \leq T_{NP}, \tag{12}$$

where T_{HBM} is the effective temperature for the rotation of the nanoparticle. We have neglected the Kapitza resistance between the nanoparticle and the liquid. Rings *et al.*[4] provide a graph of the variation of δT_{HBM} as a function of the parameter $\sqrt{1-(D/L)^2}$ of the GNRs. By using their graph and considering the average dimensions of our GNRs ($D = 14.9\,\mathrm{nm}$ and $L = 51.7\,\mathrm{nm}$), we find:

$$T_{HBM} = T_0 + 0.65\ (T_{NP} - T_0), \tag{13}$$

where T_0 is the laboratory temperature. The effective viscosity around the heated particle follows from the well-known temperature dependence of the viscosity of water, modelled by a Vogel–Fulcher equation:[3,5]

$$\eta(T) = \eta_\infty\ exp[A/(T - T_{VF})]. \tag{14}$$

In water, $\eta_\infty =$ $0.0298376 \times 10^{-3} Pa.s$, $A = 496.889°K$, and $T_{VF} = 152.0°K$. The effective viscosity and temperature are chosen such that $\eta_{HBM} = \eta(T_{HBM})$.

Then, by using Tirado's model in eq. 1 (main text) we can write the effective rotational diffusion constant as:

$$\Theta_{HBM} = \frac{3\ k_B T_{HBM}}{\pi\ \eta_{HBM} L^3}\ (lnp - 0.662 + 0.917/p - 0.050/p^2). \quad (15)$$

By considering the size distribution of our GNRs as obtained from TEM images and different incident powers, we can calculate Θ_{HBM} and compare it with the rotational diffusion coefficient in the absence of any heating (Θ). Without any heating, the histogram of rotational diffusion coefficients Θ shows a Gaussian distribution. In the presence of heating, different GNRs will experience different effective temperatures T_{HBM} and local viscosities because of their different dimensions, aspect ratios and absorption cross sections. Therefore their rotational constants Θ_{HBM} will also vary. The histogram of rotational diffusion coefficient Θ_{HBM} displays two populations, which split apart upon increasing the incident power. Those GNRs whose resonance is close to the laser 785 nm and which have larger volumes (same population as fig. S10b) will have higher effective temperature and lower local viscosity compared to the rest of the GNRs. Therefore, they will tend to tumble faster than the rest of the population (higher Θ_{HBM}).

In order to visualize this process, we have calculated Θ_{HBM} for the whole population of our GNRs (blue circles in fig. S2a) for different powers. We apply the same powers we used in our confocal measurements: 49, 147, 262, 369 µW for 785 nm. To simplify the calculations, we consider a linearly polarized plane wave and calculate the maximal absorption cross section σ_{abs} for GNRs aligned with the polarization. However, one should remember that the GNR tumbles in 3D with respect to the electric field. To account for this, we average the absorption cross section of each GNR to $\sigma_{abs}/3$, as if heat diffusion would be slow compared to rotational diffusion. As this is not necessarily the case, we point out a new theoretical problem: anisotropic rotational hot Brownian motion, where the heating power depends

on the angle of the anisotropic particle's axis with the electric field. In the absence of a theoretical treatment of this case, we apply the approximation of the average absorption cross section. This and other effects (see hereafter) can explain the larger deviation of the two populations in the calculated histograms in comparison to the measured histograms in fig.3 in the main text.

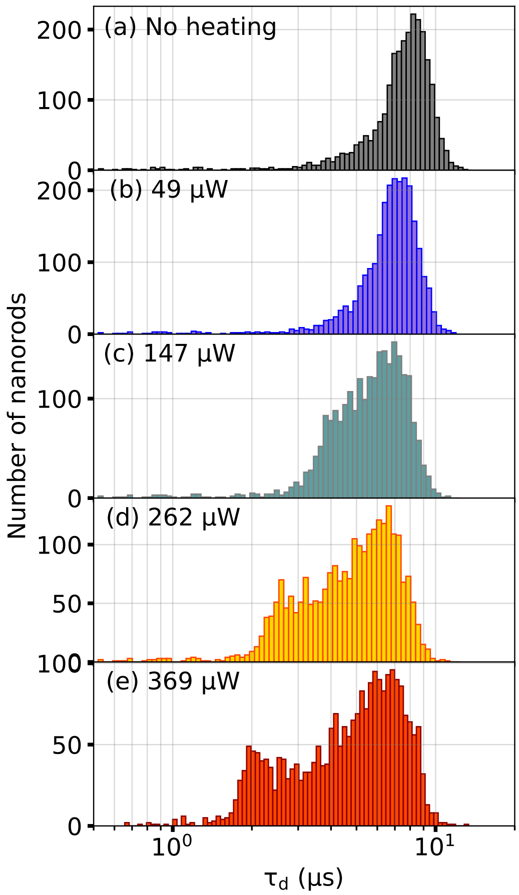

Figure S14: Calculated rotational correlation decay time by considering $\tau_d \approx \frac{1}{6\Theta}$. We have calculated Θ by Tirado's formula for the distribution of our GNRs from TEM images (fig. S2a blue circles) and considering 2.2 nm CTAB layer. **a)** Without considering any heating. **b)** By considering 49 µW incident power (785 nm as wavelength), we calculate the absorption cross section σ_{abs}, effective temperature T_{HBM} and the effective viscosity η_{HBM} for each GNR and finally we calculate Θ_{HBM} by equation 15 with considering 2.2 nm CATB layer. Then, we plot the histograms of $\tau_d \approx \frac{1}{6\Theta}$. **c)** Histogram of τ_d for 147 µW as the incident power. **d)** Histogram of τ_d for the incident power of 262 µW. **e)** Histogram of rotational diffusion times τ_d for the incident power of 369 µW. A second population of diffusion times appears by increasing the incident power, which corresponds to large and resonant GNRs which are hotter than the rest.

S6.2 Measured rotational HBM time trace

In this subsection, we provide the full traces of the excerpts shown in the main text (fig.3 i and j). On average, the amplitudes of the events in fig. S15 are higher than for the events in fig. S16 and the durations of the sub-bursts in fig. S15 are shorter than for the sub-bursts of fig. S16. As shown in fig.3h in the main text, the two events in fig. S15c and fig. S16c belong to two populations of GNRs with different rotational diffusion times τ_d. We assign this deviation to a higher absorption cross section of these GNRs.

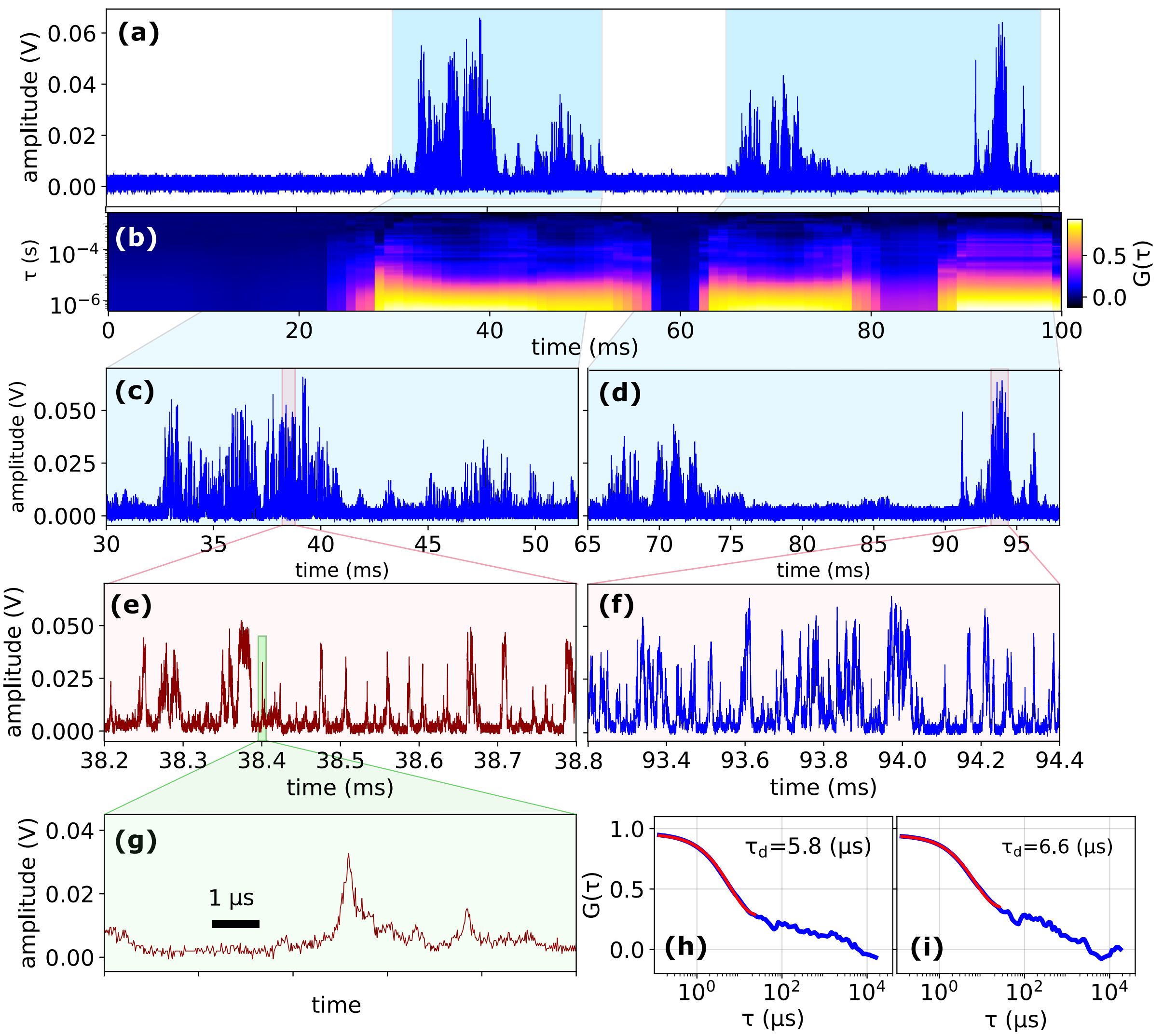

Figure S15: An example of measured Rotational Hot Brownian Motion for a large, resonant GNR. **a)** 100 ms scattering time trace. **b)** Intensity graph of the autocorrelation over the scattering trace in (a). **c, d)** Part of the two events that show fast and high amplitude fluctuations. **e, f)** Zoom-in on the events shown in (c and d), showing the fast sub-bursts with high time resolution. Part (e) is the same excerpt that is shown in fig.3j in the main text, with 369 µW as incident power. **g)** Zoom-in on one of the fast sub-bursts that shows the high time resolution of our measurements. The rise time in this sub-burst is less than 200 ns, and it is not broadened by insufficient sampling. **h, i)** Autocorrelation of the two highlighted events in (a) and a single exponential decay (red) as a fitting function which shows 5.8 µs and 6.6 µs decay times for the first and the second events, respectively.

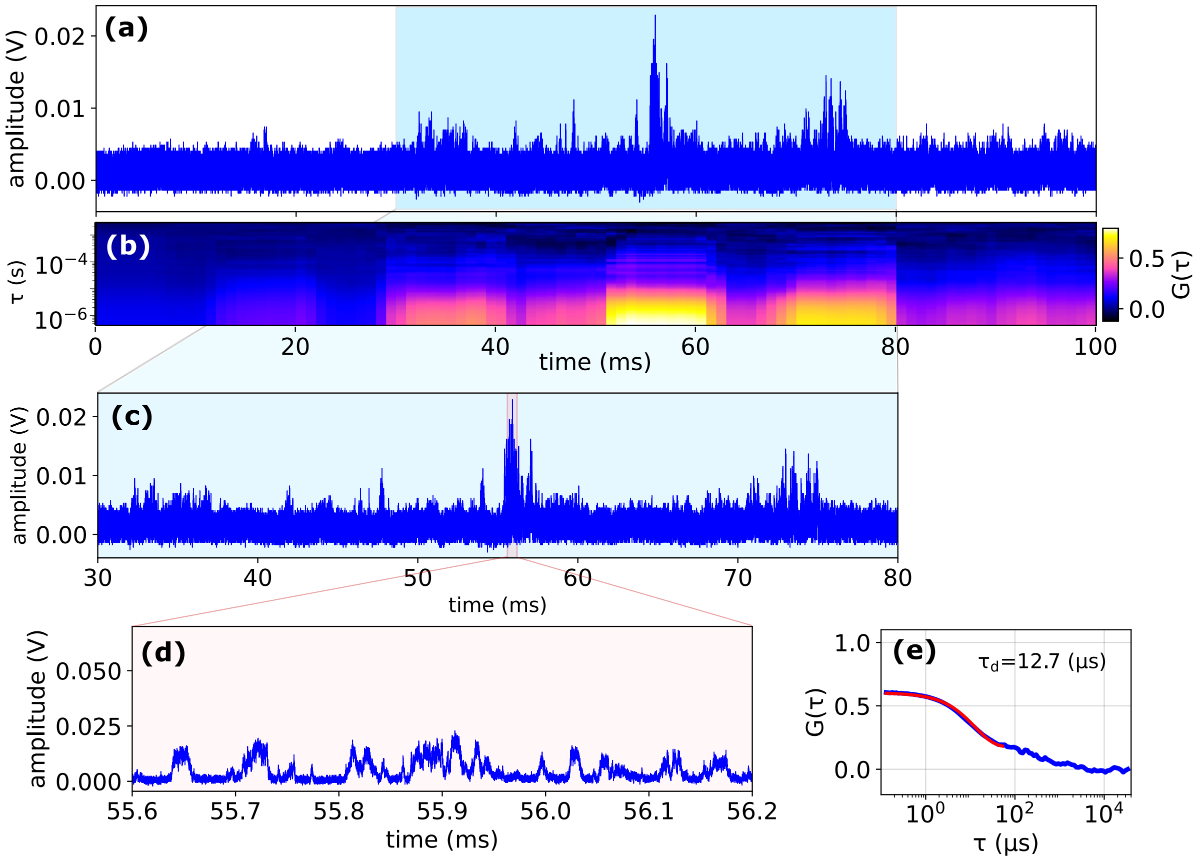

Figure S16: An example of rotational diffusion with high laser power (369 µW) for a GNR which is small or not completely resonant. **a)** Scattering time trace which shows lower amplitude fluctuations in comparison to fig. S15a. **b)** Sliding-window autocorrelation of the trace in (a). **c)** Zoom-in to part of the event showing several sub-bursts. **d)** Further zoom-in on the sub-bursts reveals slower fluctuations in comparison to fig. S15e. The Y-axis has been plotted on the same scale as in fig. S15e. This trace is also shown in fig.3i in the main text. **e)** Autocorrelation of the whole event with a single exponential fitted function (red) which shows 12.7 µs as decay time, i.e., slower rotational diffusion than in fig. S15.

S6.3 Translational hot Brownian motion

Whereas many rotational diffusion sub-events are sampled during the passage of a nanorod in the confocal volume, only one translational diffusion event is recorded for each nanorod. Therefore, much better statistics can be accumulated in rotation, whereas translational times are subject to a large statistical distribution depending on the specific trajectory of each rod in the confocal volume. Moreover, fluctuations due to rotational diffusion are in principle

independent of the optical probing geometry defined by the confocal volume. To illustrate this drawback of translational diffusion, we have acquired histograms of translational times from our events (one event per GNR). We have extracted the translational diffusion times from the slow component of our single-event autocorrelation (only events not overlapping with either the begin or the end of our time traces were considered). Expectedly, we find that the distribution of translational diffusion times is relatively much broader than that of rotational times, particularly at high intensities (see fig. S17). By comparing the translational diffusion histograms of fig. S17 with the rotational diffusion histograms of fig. 3 of the main text, we see that rotational diffusion is more sensitive than translational diffusion to heating effects.

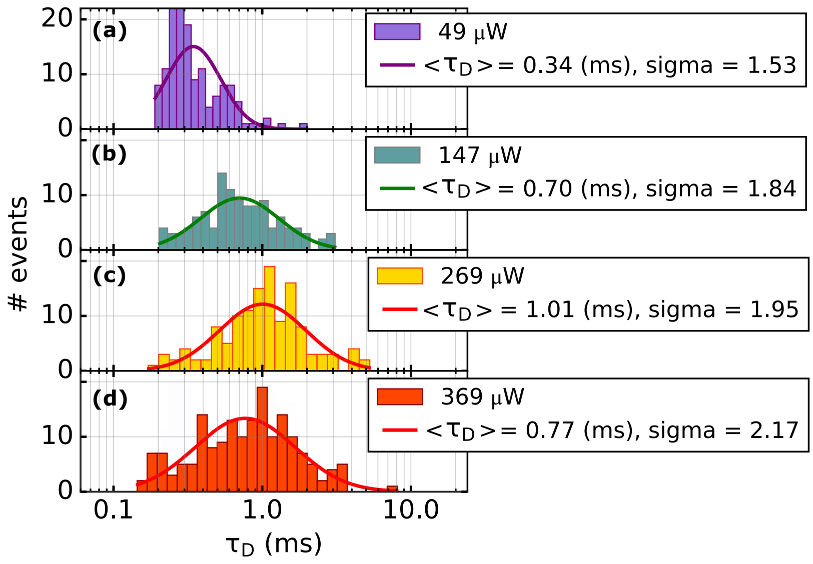

Figure S17: Power dependence of the translational diffusion times extracted from the autocorrelation of single events, fitted by log-normal distributions (solid lines). Note the large width of these distributions and the reduced information content compared to the rotational diffusion histograms. The measurements have been done for four different powers (same as fig. 3 in the main text): **a)** 49 μW, **b)** 147 μW, **c)** 269 μW and **d)** 369 μW.

S7 Optical torque

To check the effect of forces applied by the laser onto our GNRs and their possible effect on their Brownian rotational diffusion, we performed calculations considering the average

dimension of our GNR (D=14.9 nm and L=51.7 nm).

The optical forces and torques acting on the nanoparticles in an electric field can be calculated from the polarizability of the nanoparticle. We approximate our GNR as an ellipsoid. The polarizability of an ellipsoid is given by:[6]

$$\alpha_{\parallel,\perp} = 3\epsilon_0\epsilon_m V_p \frac{\epsilon - \epsilon_m}{3\epsilon_m + 3L(\epsilon - \epsilon_m)}, \tag{16}$$

where $\alpha_{\parallel}$ and $\alpha_{\perp}$ are the polarizability of the ellipsoid when the applied field is parallel and perpendicular to the longitudinal axis of the ellipsoid, respectively. V_p is the volume of the particle, ϵ_m and ϵ are the complex dielectric functions of the medium and the gold nanoparticle, respectively. $L_{\parallel}$ and $L_{\perp}$ are geometrical factors, which can be written as functions of eccentricity $e = \sqrt{1 - a^2/b^2}$, where the ellipse's half-axes a and b have been replaced by the length L and diameter D of the nanorod:

$$L_{\parallel} = \frac{1 - e^2}{e^2}\,(-1 + \frac{1}{2e}ln\frac{1+e}{1-e}) \tag{17}$$

$$L_{\perp} = \frac{1 - L_{parallel}}{2}. \tag{18}$$

The anisotropic GNR polarizability leads to an optical potential energy in an electric field with amplitude E_0, which depends on the orientation of the GNR longitudinal axis with respect to the electric field:

$$U(\Theta) = -\frac{1}{4}Re[\Delta\alpha]E_0^2\ cos^2\theta, \tag{19}$$

where $Re[\Delta\alpha]$ is real part of the difference between longitudinal and transverse polarizabilities of the GNR ($\alpha_{\parallel} - \alpha_{\perp}$).

By considering the average dimension of our GNRs (D=14.9 nm and L=51.7 nm) and simulating the absorption cross section σ_{abs} by MNPBEM toolbox[1] in MATLAB, we calculate

the effective temperature T_{HBM} in equation 13 and the potential $U(\Theta)$ in equation 19. We have done the calculation for the 4 different powers used in the manuscript. The results are shown in fig. S18. We find that the maximum optical energy is around 8×10^{-21} N m at the highest power 369 µW (red) and for the case that the GNR is aligned along the electric field. At the effective temperature (around 420 K) found for this case this potential is 1.38 times higher than k_BT. That means the applied torque can have a significant effect on the Brownian rotational motion of a large and resonant rod.

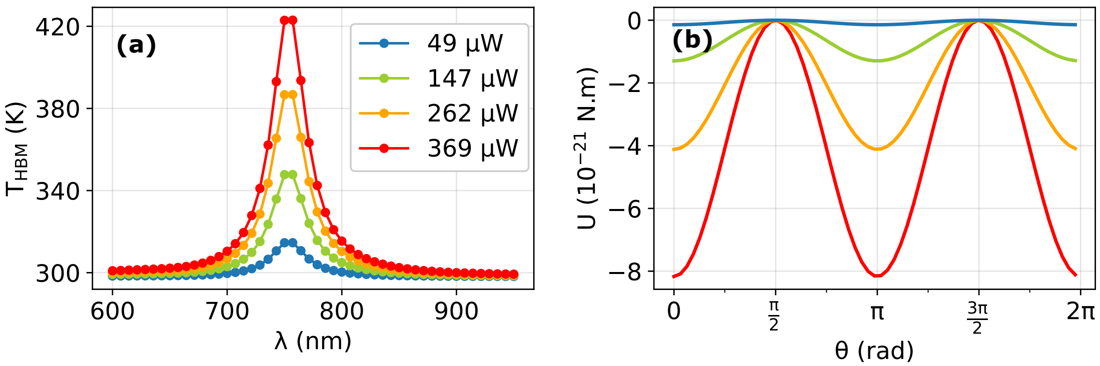

Figure S18: **a)** Effective temperature T_{HBM} of a GNR with 14.9 nm as diameter and 51.7 nm length as a function of incident wavelength, which shows a peak at 750 nm (average resonance of our GNRs, see fig. 1h). Different colors correspond to 4 different powers: 49 (blue), 147 (green), 262 (orange) and 369 µW (red). By increasing the illumination intensity, the effective temperature increases, especially at around the resonance wavelength. **b)** Optical potential of the GNR in an applied electric field as a function of the angle θ (the angle between the GNR main axis and the incident electric field). The colors correspond to the different powers mentioned in (a). The minimum of the potential energy is obtained in the case of a positive real polarizability for the highest power (red) and at θ=0, π and 2π. It is around 8×10^{-21} N m for an average resonant GNR. If we compare it with the value of k_BT at this temperature (that is corresponding to the Brownian motion) which is $k_BT = (1.38 \times 10^{-23})420 = 5.79 \times 10^{-21}$ N m, we find that maximum absolute potential for on the resonance GNR is 1.38 times more than k_BT. It means the applied torque has a significant effect on the rotational Brownian motion at our highest excitation power (369 µW).

S8 Rotational diffusion in PVA solutions

Binding affinity of PVA to the GNRs at very low concentrations

The adsorption process can be described by Langmuir's adsorption (isotherm) model[7] as

a reversible chemical process:

$$P + S \rightleftharpoons SP, \tag{20}$$

where P is the adsorbate molecule, S is an empty adsorption site and SP the same site with the adsorbed species. By considering a and d as adsorption and desorption rates, respectively, we write the fraction of occupied surface sites as:

$$\frac{[SP]}{[S_0]} = \frac{a[P]}{d + a[P]}, \tag{21}$$

where closed brackets are the concentrations or populations of each species. $[S_0] = [S] + [SP]$ is the total population of adsorption sites. We assume that the hydrodynamic volume of the diffuser, V_H, increases linearly upon binding polymer chains. By assuming that the rotational correlation decay time (τ_d) scales linearly with the volume of the object, which itself varies linearly with the amount of polymer bound, we can write:

$$\tau_d = \tau_{d\,0} + \Delta\tau_d \, \frac{[SP]}{[S_0]}, \tag{22}$$

where $\tau_{d\,0}$ is the decay time of the rotational component of the autocorrelation function of the GNR without any polymer bound to it. Consequently, the y axis of the plot in fig. 4d in the main text is proportional to the ratio of occupied sites to the total number of sites $[SP]/[S_0]$. It means that, by increasing the concentration of PVA, the number of occupied sites increases and at very high concentration, all the sites will be occupied by PVA.

For the lowest concentrations of PVA in our measurement (62 and 125 ppb, fig.4 a and b in the main text) by considering the population of the fast decay times as the free rods and the second population as the occupied ones and ignoring the heterogeneity of binding sites, we can approximately write:

$$\frac{[SP]}{[S_0]} \approx \frac{p_1}{p_0 + p_1}, \tag{23}$$

where p_1 is the probability of finding a GNR occupied by at least one polymer coil bound and p_0 is the probability of detecting a GNR without any polymer. We assign the two Gaussian distributions in the histograms of fig.4 a and b to the populations of free rods and occupied rods. p_0 and p_1 are proportional to the areas of these histogram components.

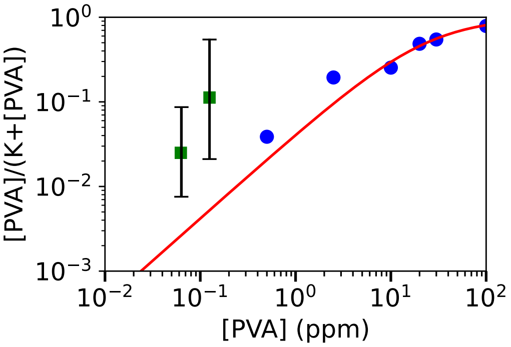

Figure S19: Fraction of adsorption sites with bound PVA as a function of the PVA concentration in a double-logarithmic plot. The blue dots are the points of fig.4d in the main text, with the fitted Langmuir isotherm in red. The two green squares with error bars are estimated from the histograms at extremely low PVA concentrations, 62 and 125 ppb. The fraction of occupied sites has been estimated approximately from the histograms of fig.4 a and b in the main text. These data show a qualitative deviation from the expected Langmuir isotherm curve (red) at this concentration, which we interpret as a much higher affinity for the first occupied sites in comparison to other sites.

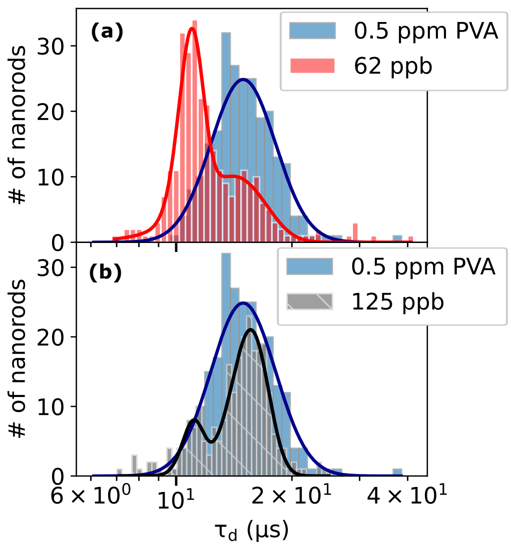

Figure S20: Histograms of decay times τ_d for different concentrations of PVA: 0.5 ppm (blue), 62 ppb (red) and 125 ppb (gray). **a)** Shows an overlap between the second population of the red histogram and the blue one, which is corresponding to the GNRs with one polymer coil bound to them. **b)** There is a big overlap between the blue histogram and the gray one which shows a big ratio of the GNRs in the gray histogram are corresponding to the rods with at least one polymer coil on them.

By considering a persistence length of 3Å for PVA, and a Flory exponent of 0.6, we estimate the radius of each polymer coil to 37 nm on average.

Upon adsorption of a PVA chain (molecular weight: 125 000 g/mol) we expect a redshift on the order of few nm similar to the values obtained by P. Zijlstra et al. for Streptavidin-RPE (300 kDa, max. 2 nm shift).[8] According to sect. S5, the LSPR changes in the range of a few nanometers do not affect the rotational diffusion coefficient.

We also investigate the distribution of translational diffusion times by extracting the decay time of slow component of autocorrelations τ_D. We compare the results for three measurements: GNR without PVA, GNR with 0.5 ppm PVA and 100 ppm PVA, and present them in fig. S21. As discussed in sect. 6.3, we find broader histograms than for rotational diffusion. Moreover, these histograms overlap considerably, which confirms that rotational diffusion is a better tool to study binding of biomolecules to GNRs.

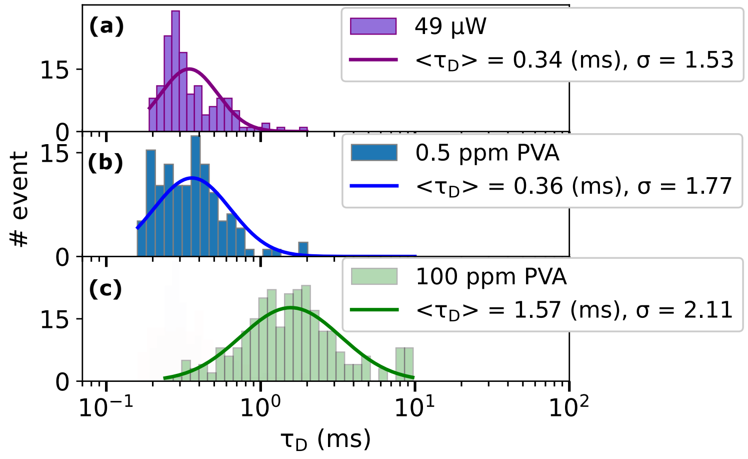

Figure S21: Histograms of decay times of the translational component of the scattering autocorrelation τ_D for **a)** GNRs without PVA, **b)** GNRs with 0.5 ppm PVA and **c)** GNRs with 100 ppm PVA.

S9 Observation of protein adsorption

In order to confirm the possible application of our method in a bio-sensing context we compare the rotational decay times of gold nanorods in the absence and the presence of 10 µM Bovine Serum Albumin (BSA, molecular mass 66 kDa). We find that the τ_d histogram obtained in presence of BSA is significantly broader than its counterpart obtained in the absence of BSA, confirming the observation of BSA adsorption to some of the NRs. (see Fig. S18)

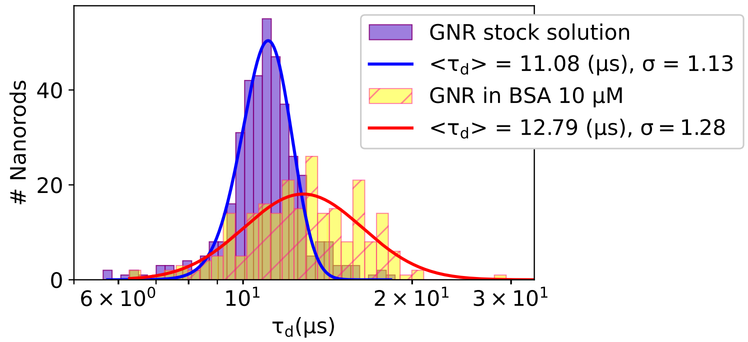

Figure S22: Histograms of decay times τ_d measured in the absence (purple) and the presence of BSA (yellow). Solid lines indicate the respective fits to Gaussian (blue) and lognormal(red) distributions.

The mean value of the diffusion times in presence of BSA increases by 1.71 μs (16 %) indicating an increase of hydrodynamic volume by 16%, i.e., by $\approx 1100\,\mathrm{nm}^3$. From this value we can approximate the average number of BSA molecules adsorbed to GNR (assuming BSA molecule as spheres with diameters of 7 nm and volumes of $\approx 180\,\mathrm{nm}^3$) as $N_{BSA} \approx 6$.

References

(1) Hohenester, U.; Trügler, A. MNPBEM–A Matlab Toolbox for the Simulation of Plasmonic Nanoparticles. *Computer Physics Communications* **2012**, *183*, 370–381.

(2) Baffou, G. *Thermoplasmonics: Heating Metal Nanoparticles Using Light*, 1st ed.; Cambridge University Press, 2018; pp 81–96.

(3) Rings, D.; Schachoff, R.; Selmke, M.; Cichos, F.; Kroy, K. Hot Brownian Motion. *Physical review letters* **2010**, *105*, 090604.

(4) Rings, D.; Chakraborty, D.; Kroy, K. Rotational Hot Brownian Motion. *New Journal of Physics* **2012**, *14*, 053012.

(5) Fulcher, G. S. Analysis of Recent Measurements of the Viscosity of Glasses. *Journal of the American Ceramic Society* **1925**, *8*, 339–355.

(6) Bohren, C. F.; Huffman, D. R. *Absorption and Scattering of Light by Small Particles*; WILEY-VCH, 1998.

(7) Langmuir, I. The Adsorption of Gases on Plane Surfaces of Glass, Mica and Platinum. *Journal of the American Chemical society* **1918**, *40*, 1361–1403.

(8) Zijlstra, P.; Paulo, P. M. R.; Orrit, M. Optical Detection of Single Non-Absorbing Molecules Using the Surface Plasmon Resonance of a Gold Nanorod. *Nature Nanotechnology* **2012**, *7*, 379–382.
